# Fast self-navigated wall shear stress measurements in the murine aortic arch using radial 4D-phase contrast cardiovascular magnetic resonance at 17.6 T

**DOI:** 10.1186/s12968-019-0566-z

**Published:** 2019-10-14

**Authors:** Patrick Winter, Kristina Andelovic, Thomas Kampf, Fabian Tobias Gutjahr, Julius Heidenreich, Alma Zernecke, Wolfgang Rudolf Bauer, Peter Michael Jakob, Volker Herold

**Affiliations:** 1 Experimental Physiks V, University of Würzburg, Am Hubland, 97074 Würzburg, Germany; 2 Medical Clinic and Policlinic I, University Wuerzburg, Oberdürrbacher Straße 6, 97080 Würzburg, Germany; 3 Institute for Experimental Biomedicine, University Hospital Würzburg, Josef-Schneider-Straße 2, 97080 Würzburg, Germany; 4 Diagnostic and Interventional Neuroradiology, University Hospital Würzburg, Josef-Schneider-Straße 11, 97080 Würzburg, Germany

**Keywords:** 4D flow, WSS, OSI, Self-navigation, Mouse, Aortic arch

## Abstract

**Purpose:**

4D flow cardiovascular magnetic resonance (CMR) and the assessment of wall shear stress (WSS) are non-invasive tools to study cardiovascular risks in vivo. Major limitations of conventional triggered methods are the long measurement times needed for high-resolution data sets and the necessity of stable electrocardiographic (ECG) triggering. In this work an ECG-free retrospectively synchronized method is presented that enables accelerated high-resolution measurements of 4D flow and WSS in the aortic arch of mice.

**Methods:**

4D flow and WSS were measured in the aortic arch of 12-week-old wildtype C57BL/6 J mice (*n* = 7) with a radial 4D-phase-contrast (PC)-CMR sequence, which was validated in a flow phantom. Cardiac and respiratory motion signals were extracted from the radial CMR signal and were used for the reconstruction of 4D-flow data. Rigid motion correction and a first order *B*_0_ correction was used to improve the robustness of magnitude and velocity data.

The aortic lumen was segmented semi-automatically. Temporally averaged and time-resolved WSS and oscillatory shear index (OSI) were calculated from the spatial velocity gradients at the lumen surface at 14 locations along the aortic arch. Reproducibility was tested in 3 animals and the influence of subsampling was investigated.

**Results:**

Volume flow, cross-sectional areas, WSS and the OSI were determined in a measurement time of only 32 min. Longitudinal and circumferential WSS and radial stress were assessed at 14 analysis planes along the aortic arch. The average longitudinal, circumferential and radial stress values were 1*.*52 *±* 0*.*29 N/m^2^, 0*.*28 *±* 0*.*24 N/m^2^ and *−* 0*.*21 *±* 0*.*19 N/m^2^, respectively. Good reproducibility of WSS values was observed.

**Conclusion:**

This work presents a robust measurement of 4D flow and WSS in mice without the need of ECG trigger signals. The retrospective approach provides fast flow quantification within 35 min and a flexible reconstruction framework.

**Electronic supplementary material:**

The online version of this article (10.1186/s12968-019-0566-z) contains supplementary material, which is available to authorized users.

## Background

Cardiovascular diseases such as aortic valve disease, aneurysms and atherosclerosis are responsible for almost 25% of deaths in the US [1]. The hemodynamic environment is described by parameters such as flow and wall shear stress (WSS) and plays an important role in the development of these diseases [2, 3], since the complex vascular geometry and the pulsatile flow in the arterial system lead to regionally different flow characteristics and thus spatial and temporal changes in shear forces acting on the vessel wall [4]. The WSS is proportional to the spatial velocity gradient at the vessel wall and can therefore be assessed from the measured velocity fields. It is a vector quantity, which is expressed in N/m^2^ [5]. Shear stress can be dissected into a longitudinal part in parallel to the vessel and along the preferred flow direction and a circumferential component, which is perpendicular to the longitudinal component. In addition, normal stress components can occur when blood flow components pointing towards the surface normal of the vessel wall are present [6]. As the normal component is pointing in radial direction towards the center of the vessel [7], this component will be referred to as “radial stress” in the following. A further important parameter associated with the WSS is the oscillatory shear index (OSI), which describes the temporal variability of the WSS waveform. Both low WSS and high OSI values are potential markers for the formation of plaques in the aorta and other vessels [8]. WSS and OSI can be estimated non-invasively by measuring the flow velocities with phase contrast (PC) - cine cardiovascular magnetic resonance (CMR). Two-dimensional acquisitions provide localized information regarding hemodynamic forces and flow rates [9]. However, the difficulty in localizing a slice perpendicular to the flow direction in curved vessels such as the aortic arch can lead to experimental errors, especially near the aortic branches. Thus, a three-dimensional acquisition is needed to characterize the complete hemodynamic environment throughout the aortic arch. Conventional electrocardiogram (ECG)-triggered 3D measurements of flow and WSS however, are limited in spatiotemporal resolution due to long measurement times [7, 10]. This usually leads to an underestimation of WSS [5]. Measurements in mice are particulary challenging due to the small scale of the murine aorta and the high demands on animal handling, as unstable heart rates can impede the assessment of accurate flow waveforms. This becomes even more problematic at ultra-high-field strengths, where ECG-based navigation can become unreliable due to magnetohydrodynamic effects and interferences with the fast switching of the imaging gradients [11, 12]. In recent studies, the use of radial acquisitions for flow measurements in mice have been investigated [9, 13]. Radial trajectories are less prone to flow artifacts and provide an intrinsic cardiac motion signal, which can be used for self-gated ECG-free measurements [14]. The use of self-navigation for 3D flow measurements in the murine heart has already been investigated [15]. However, a measurement time of approximately 2 h was still needed for a complete dataset.

Time-of-flight (TOF) CMR techniques achieve a high blood-tissue contrast by exciting small image volumes using excitation pulses with large flip angles and small repetition times. This leads to a strong signal enhancement for the inflowing blood and a strong suppression of the static background. As a result, large undersampling factors are possible with 3D radial acquisitions [16].

In this work we propose an advanced method based on a self-navigated 3D radial PC-cine FLASH (Fast Low Angle SHot) acquisition, which exploits the inflow effect in order to achieve high resolution (isotropic 100 μm) flow measurements in the murine aortic arch in only 32 min. With this method, 3D flow velocities, aortic cross sectional areas, longitudinal, circumferential and radial stress components and the oscillatory shear index were determined.

## Methods

### Animal handling

All studies were conducted according to a protocol approved by the Institutional Animal Care and Use Committee. Female wild-type (WT) C57BL/6 J mice (*n* = 7) were obtained from Charles River Laboratories (Sulzfeld, Germany) and were studied at an age of 12 weeks. Mice were anesthetized with 4% isoflurane in 2.0 Vol.% oxygen (2 L/min), applied by a nose cone and were positioned vertically (head up). For cardiac and respiratory monitoring during the measurement, a pressure- sensitive pneumatic balloon (Graseby Medical Limited, Watford, United Kingdom) was placed between the inner radio frequency (RF) resonator wall and the murine thorax. The pressure signal from the balloon was transformed into an electrical signal by a pressure transducer (24PCEFA6 D, Honeywell S&C, Golden Valley, Minnesota, USA) and was amplified and processed in real-time by a custom-built ECG unit [17]. Due to the small inner diameter of the gradient insert and the RF coil, core body temperature could be maintained at physiological 37 *°*C during CMR-measurements by adjusting the temperature of the gradient cooling unit.

### Data acquisition

**Fig. 1:**
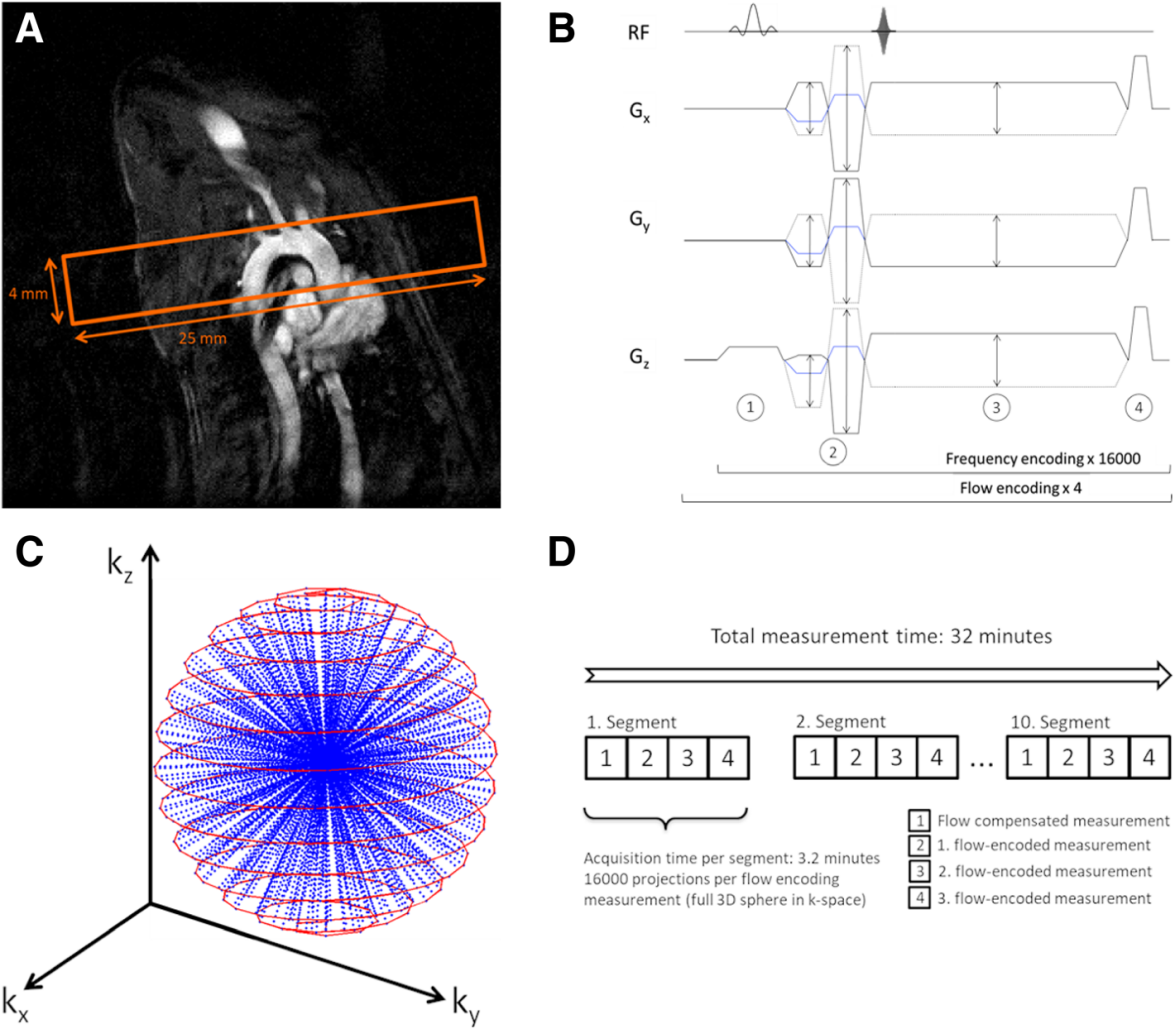
**A** Slice positioning: A 3D image volume (25 *×* 25 *×* 4 *mm*^3^) was positioned perpendicular to the aortic arch. **B** Radial Phase-Contrast (PC) cine sequence. 1. Slice excitation with a Sinc pulse. 2. Bipolar dephase / rephase gradients for flow compensation. Flow encoding is performed simultaneously with the dephase gradients (blue lines). 3. 3D frequency encoding with an echo asymmetry of 10%. 4. After the readout constant gradient spoiling is applied in all 3 directions. **C** Used spatial encoding scheme: Spherical 3D k-space data points were sampled using a spiral-shaped trajectory for the radial projections. **D** To increase robustness the measurement (*ns* = 1*.*6 x 10^5^ radial projections) was segmented into 10 smaller subsets, which were applied in a sequential order. Each segment consists of 4 flow-encoding measurements with 1*.*6 x 10^4^ projections, respectively, which are measured one at a time. Each measurement covers a full 3D sphere in k-space

Measurements were performed with a 17.6 T vertical-bore small animal MR system (Bruker Avance 750 WB, Bruker BioSpin MRI GmbH, Rheinstetten, Germany, operated with Paravision 4.0) with a 1 T/m gradient system (diameter: 40 mm) and a custom-built single-channel transmit-receive electromagnetic (TEM) resonator (inner diameter: 24 mm). To localize the position of the aortic arch, balloon-triggered axial and longitudinal 2D-cine FLASH measurements were acquired. Subsequently, retrospective flow measurements were performed with a radial PC-FLASH-sequence (see Fig. 1B) in a 3D slab perpendicular to the aorta (image volume: 25 *×* 25 *×* 4 *mm*^3^, see Fig. 1A). Spatial encoding was performed with a 3D radial trajectory with an angular density optimized for the anisotropic field of view [18], which was calculated with an open source tool box [19]. For flow-encoding a balanced 4-point flow-encoding scheme [20] with an encoding velocity of *v*_ENC_ = 125 cm/s was used. Each flow-encoding step consists of a read-out with 1.6 x 10^5^ radial projections (140 read-out points, TR = 3 ms) covering a 3D-sphere in k-space (Fig. 1C). A flip angle of 15° was used in order to achieve high blood-tissue contrast. To minimize artifacts caused by off-resonances and signal dephasing due to accelerated flow, the echo time was set to 1.1 ms. In order to guarantee such a short echo time, it was necessary to design the amplitudes of the dephase gradients in a way that the gradient echo occurs at *t*_*E*_ = 0*.*1 *× t*_*acq*_, where *t*_*acq*_ is the acquisition time (Fig. 1B). To further increase robustness of data acquisition, the measurement was segmented into 10 subsets, each consisting of 1*.*6 *×* 10^4^ projections (each covering a full 3D sphere in k-space) and 4 flow-encoding steps (Fig. 1D), which were acquired one at a time.

This kind of segmentation allows repetition of corrupted data sets in case of disturbances, e.g. caused by instabilities of the heart rate. Acquisition time of one subset was 3.2 min, leading to a total measurement time of 32 min for a full 4D flow protocol.

### Phantom measurements

The stability of the 4D flow-encoding sequence was tested in a phantom consisting of a flow pump (MPC-Z V1.10, ISMATEC, Cole-Partner GmbH, Wertheim, Germany) with constant flow and adjustable flow values (max flow: 50 ml/s) and a silicone tube (ø = 6 mm). Flow was measured at 10 different flow values (15.00 ml/s - 26.25 ml/s in equidistant steps) with the protocol described above using only one subset per measurement (scan time: 3.2 min per subset) and the same encoding velocity as the in vivo measurements *v*_ENC_ = 125 cm/s. Mean flow values and standard deviations were calculated over 10 slices. To prevent artifacts due to phase aliasing, phase unwrapping was applied when necessary [21]. For comparison, flow was also quantified by measuring volumetrically in liters. The reference measurement was repeated 8 times and mean values and standard deviations were determined.

### Self-navigation

All signal processing was performed with MATLAB (The Mathworks, Inc., Natick, Massachusetts, USA). For retrospective self-navigation, the magnitude value of the center k-space signal (*k* = 0) was used. First, high-frequency disturbances were removed by using a matched filter for low-pass filtering [22]. The matched filter can be interpreted as a convolution of the noisy navigator signal with a conjugated time-reversed small portion of the signal [23]. After filtering a baseline subtraction [24] was used in order to eliminate low-frequency modulations caused by respiratory motion and by the transient to the steady state.

**Fig. 2:**
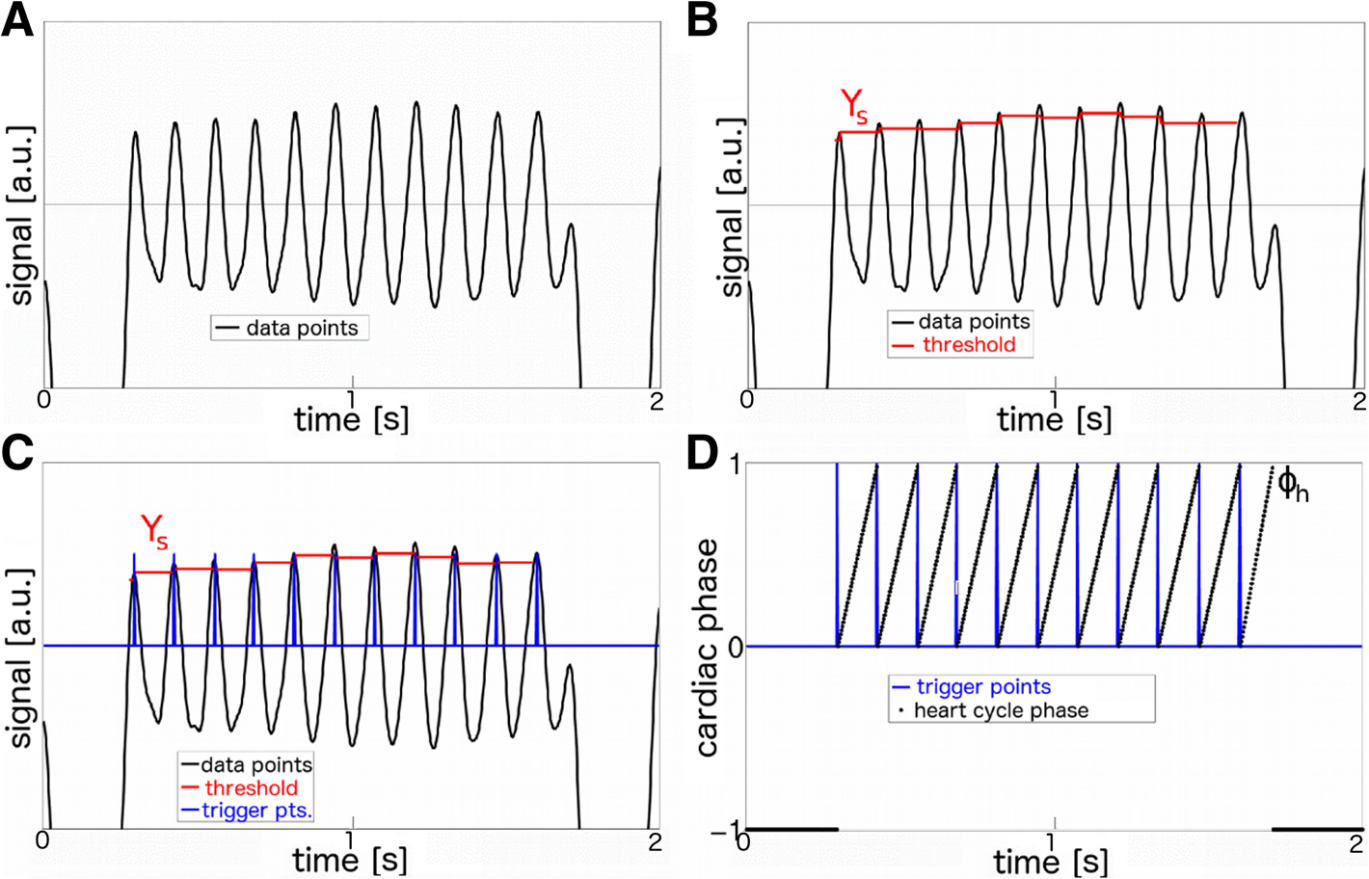
Analysis of the self-gating signal. **A** Cardiac signal. **B** Calculation of variable thresholds using a moving maximum algorithm. **C** Calculation of trigger time stamps. The first value above the thresholds is defined as trigger point. **D** Calculation of relative heart cycle phases for each time stamp. Data points detected during respiration are assigned to a phase value Φ_*h*_ = − 1 (this algorithm was presented first by our group in [13])

Trigger points and breath gating intervals were determined with variable thresholds (Fig. 2A-C). Using a linear assignment, each read-out was afterwards allocated to a value between 0 and 1, corresponding to a phase in the cardiac cycle (Fig. 2D). For removal of corrupted data points due to respiratory motion, the time average of the trigger point intervals (i.e., the mean cardiac period) was calculated for all 40 subsets, respectively. Only trigger point intervals lying in a *±* 4 *×* TR interval (*±*12 ms) window around the temporal average were accepted for reconstruction. For respiratory gating data points during inspiration were assigned to a cardiac phase value of −1. For reconstruction, the read-outs were divided into 20 selection intervals, corresponding to 20 cardiac phase intervals. For each selection interval, the associated projections were combined and an image was reconstructed using a nonuniform fast Fourier transform (NUFFT) with an open source software toolbox [25, 26]. In this manner a set of four 3D-cines (one flow-compensated cine and 3 flow-encoded cines) with 20 frames and an isotropic spatial resolution of 100 μm, respectively, was reconstructed.

### Off-resonance correction

At high magnetic field strengths, *B*_0_ offsets and field gradients cause deviations of the radial trajectory, which can lead to severe blurring artifacts in the reconstructed images. To remove these artifacts, an additional flow compensated radial 3D FLASH measurement with two different echo times was performed in the same field of view (FOV) (*t*_*E*1_ = 1.3 ms, *t*_*E2*_ = 2.3 ms total measurement time: 3.2 min, spatial resolution: isotropic 100 μm). A 3D off-resonance map was calculated from the phase differences between the two images [27]:

1 $$ \Delta f\left(x,y,z\right)=\frac{\phi_2-{\phi}_1}{2\pi \cdot \left({t}_{E1}-{t}_{E2}\right)}, $$

where *t*_*E*1*,*2_ and *φ*_1*,*2_ are the echo times and phases of the two images. Assuming only a global field offset and constant field gradients and neglecting local field inhomogeneities, the spatial dependent off-resonance frequencies can be approximated as:

2 $$ \Delta f\left(x,y,z\right)\approx \Delta {f}_0+\alpha \cdot x+\beta \cdot y+\delta \cdot z. $$

To remove blurring artifacts induced by a global frequency offset ∆*f*_0_, the frequency value from the center of the FOV (*x = y = z =* 0) was taken and used for a phase correction of the MR signal:

3 $$ {S}^{\prime }(t)=S(t)\cdot {e}^{i2\pi \cdot \Delta {f}_0t}, $$

where *S*(*t*) denotes the raw uncorrected MR signal. To also correct deviations of the k-space trajectory caused by global field gradients, the corrected trajectory ${\overrightarrow{k}}^{\prime }(t)$ used for re-gridding was calculated with:

4 $$ {\overrightarrow{k}}^{\prime }(t)=\overrightarrow{k}(t)+\left(\begin{array}{c}\alpha \\ {}\beta \\ {}\delta \end{array}\right)t, $$

where ${\overrightarrow{k}}^{\prime }(t)$ denotes the undistorted radial trajectory. *α*, *β* and *δ* were determined through linear fits of the off-resonance map.

### Rigid motion correction

Due to the vertical setup of the MR scanner, a slight slipping and shifting of the mouse was sometimes observed during the measurement. Since this would lead into undesired motion artifacts such as blurring and phase subtraction errors, a rigid motion correction was applied prior to the cine reconstructions.

**Fig. 3:**
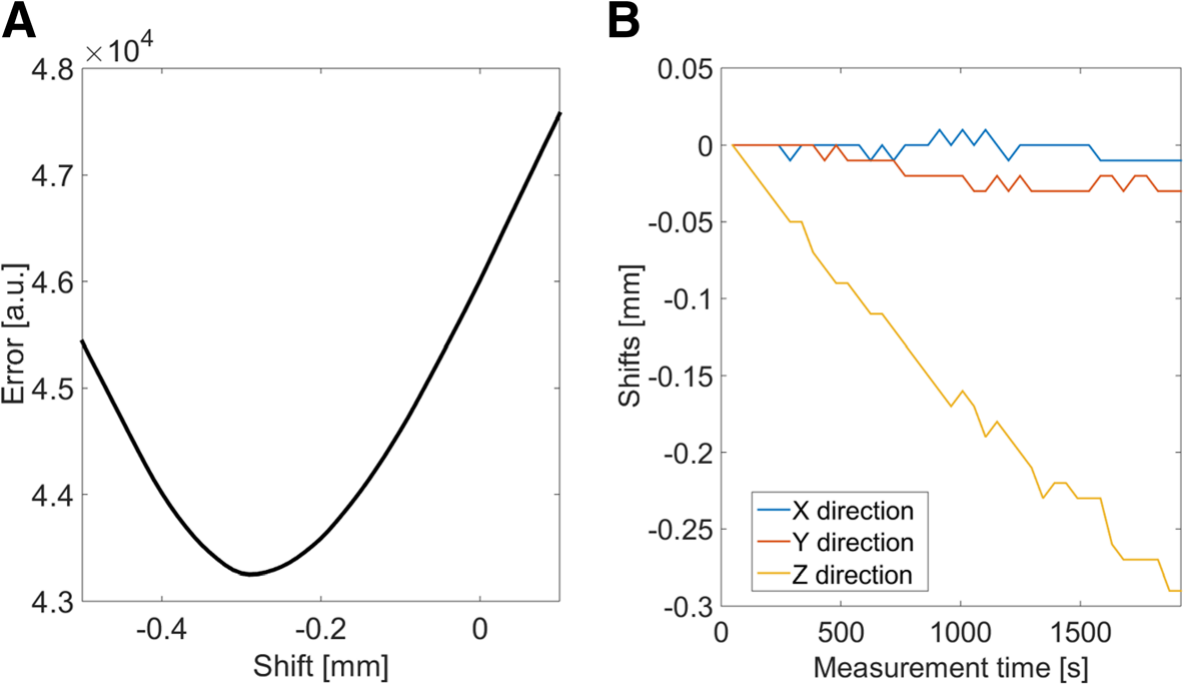
**A** Exemplary result of a shift measurement (z-direction). Each image I_n_ was shifted on a 0*.*01 mm grid and compared with the first image, *I*_1_. The optimum shift value corresponds to the minimum error between the reference and the shifted image. **B** Measured shifts in all directions as a function of time for an exemplary mouse. The shift values were determined on a time base of 48 *s*

First, the 10 measurement subsets described above were used for the reconstruction of 40 time-averaged 3D-images (4 encoders times 10 measurement subsets). Using the first image *I*_1_ as reference, the shifts x, y and z were calculated for each subsequent image *I*_*n*_ in order to minimize the error between these images (Fig. 3A):

5 $$ \Psi =\underset{x,y,z}{argmin}{\left\Vert {I}_1-{T}_{x,y,z}\cdot {I}_n\right\Vert}_2^2\kern2em n=2,3,...\mathrm{40.} $$

Hereby *T*_*x,y,z*_ denotes the translation operator in respect to the image coordinates x,y and z, which needs to be applied for minimization of the error between the first and the *n − th* image. The algorithm yields shift values on a time base of 48 *s* (Fig. 3B). This information was used for a phase correction of the signal in k-space using the Fourier Shift Theorem [28] prior to the reconstruction.

### Image processing and segmentation

Depending on the slice orientation and the alignment of the aortic arch within the image volume, the phase accumulations induced by flow encoding can cause slight deformations of the waveforms of the self-gating signal. Due to these distortions, the cines of the 3 flow-encoders are sometimes temporally shifted against the flow-compensated cine. In order to correct these temporal shifts, the time-dependent image intensities averaged over one slice were compared against each other. By using cross-correlation [29], the temporal shifts were determined for each encoder and the cines were synchronized.

For segmentation of the aortic arch, an adapted version of the previously described semi-automatic segmentation technique [30] was used. This technique assumes that the segmentation of all 4 cines should in principle lead to the same number of identified pixels. By evaluating a cost function, an optimum threshold value corresponding to a minimum deviation between the flow encoding measurements can be derived. Using this technique, each slice (in *z*-direction) of the 3D cine was segmented independently. Slices near the aortic root were excluded due to strong signal cancellations induced by accelerated flow. Subsequently, the three velocity components (*v*_*x*_, *v*_*y*_, *v*_*z*_) were calculated from the phase differences between the cines. Using the segmentation data, velocity was afterwards zeroed outside the aorta and filtered with a spatial median filter with a 3-connectivity neighborhood inside the lumen [31]. The spatial median filter removes outliers of velocity values due to segmentation errors near the lumen boundaries but leaves velocity data within smooth regions inside the vessel untouched [7].

### Calculation of WSS and OSI

Assuming a Newtonian and incompressible fluid, the general form of the WSS →*τ* can be written as [6]:

6 $$ \overrightarrow{\tau}=2\eta \overset{\cdot }{\varepsilon}\cdot \hat{n}, $$

where *η* denotes the viscosity of blood, $\hat{n}$ the inward unit normal of the lumen surface and $\overset{\cdot }{\varepsilon }$ the deformation tensor:

7 $$ {\overset{\cdot }{\varepsilon}}_{ij}=\frac{1}{2}\left(\frac{\partial {v}_j}{\partial {x}_i}+\frac{\partial {v}_i}{\partial {x}_j}\right),\kern2em i,j=1,2,3. $$

Hereby *x*_*i,j*_ denotes the spatial coordinates and *v*_*i,j*_ the velocity components.

To calculate the WSS and radial stress, the PC and segmentation data were imported into Ensight (CEI systems, USA). The velocity derivatives and the surface normals were calculated directly from the 3D velocities and the isosurface of the lumen segmentation using a custom-built Python script. For the blood viscosity, a value of *η* = 0*.*04 Pas was assumed [9].

**Fig. 4:**
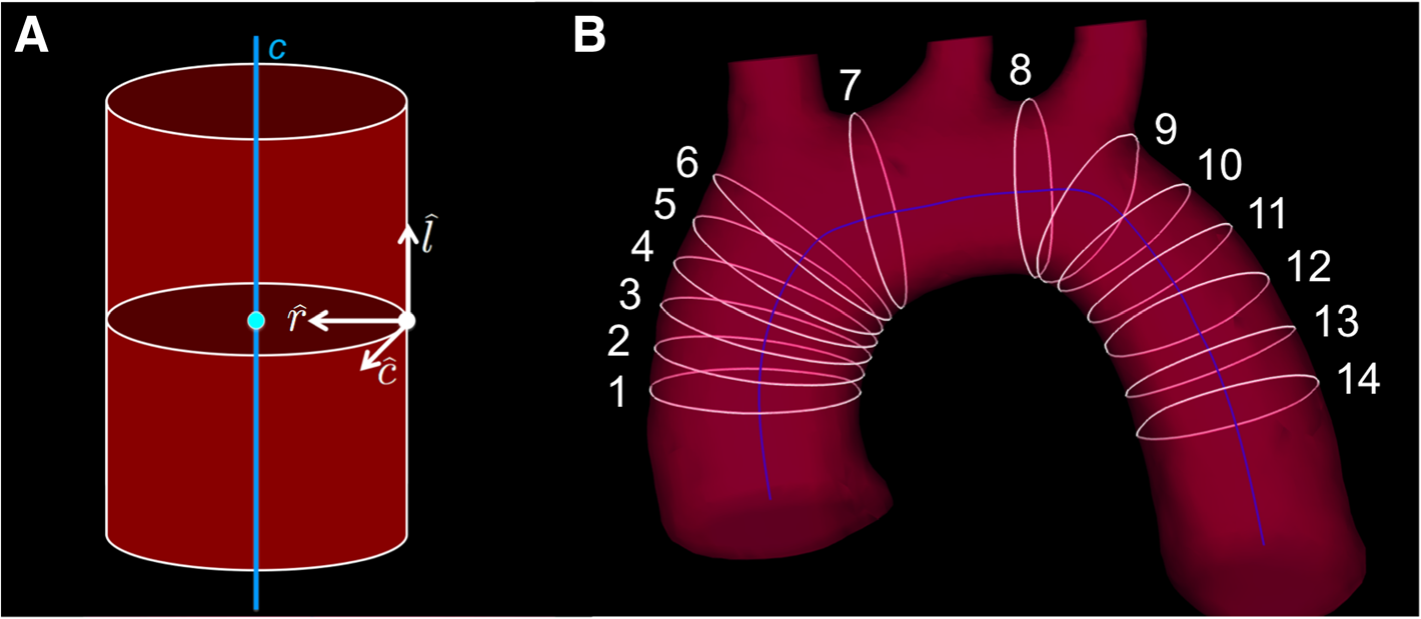
**A** To calculate the three components of the WSS, the longitudinal (*l*: in parallel to centerline, blue line), radial (*r*: pointing towards the centerline) and circumferential $\left(\hat{c}=\hat{l}\times \hat{r}\right)$ unit vectors were calculated for each point on the lumen surface, located on a ring segment perpendicular to the centerline. **B** WSS components were determined for 14 ring segments at different locations of the aorta (1–6 ascending aorta, 7–8 bifurcation area, 9–14 descending aorta). Mean and median values of all three components were calculated for each ring segment

To separate the two components of the WSS and the radial stress, a centerline of the aortic arch was calculated, as described by [7] (Fig. 4A). Ring segments perpendicular to the centerline were afterwards generated at 14 different locations of the aorta (Fig. 4B). The isosurface of the segmented lumen imported to Ensight consists of a grid with approx. 5 *·* 10^3^ nodes. For each node of the surface grid, the longitudinal ($\hat{l}$: in parallel to centerline), radial ($\hat{r}$: pointing towards the centerline) and circumferential $\left(\hat{c}=\hat{l}\times \hat{r}\ \right)$ unit vector was calculated. The WSS and radial stress can afterwards be separated using:

8 $$ {\tau}_l=\overrightarrow{\tau}\cdot \hat{l},\kern2em {\tau}_c=\overrightarrow{\tau}\cdot \hat{c},\kern2em {\tau}_r=\overrightarrow{\tau}\cdot \hat{r}. $$

Mean and median values of the three components were calculated for each ring segment and cardiac phase. In addition, temporal averaged WSS values $\left(\overline{\tau \to \left(r,\to \right)}\right)$ were derived using:

9 $$ \overline{\tau \to \left(r,\to \right)}\mid =\frac{1}{T_{RR}}{\int}_0^{T_{RR}}\overrightarrow{\tau}\left(\overrightarrow{r},t\right) dt, $$

where $\overrightarrow{\tau}\left(\overrightarrow{r},t\right)=\left[{\tau}_l(t)\kern0.5em {\tau}_c(t)\kern0.5em {\tau}_r(t)\right]$are the time-dependent WSS and radial stress components and *T*_*RR*_ is the cardiac period. To also measure the temporal variability of the WSS waveforms and the degree of oscillatory flow, the OSI was calculated from the time dependent stress values using [5]:

10 $$ \mathrm{OSI}=\frac{1}{2}\left(1-\frac{\mid {\int}_0^{T_{RR}}\overrightarrow{\tau}\left(\overrightarrow{r},t\right) dt\mid }{\int_0^{T_{RR}}\mid \overrightarrow{\tau}\left(\overrightarrow{r},t\right)\mid dt}\right). $$

No changes in the direction of the stress over time result in a minimal OSI value (OSI = 0). In contrast, when strong periodic variations and sign changes occur, e.g. caused by recirculative flow during the diastolic cardic phase, the integral value nears itself to the limit ${\int}_0^{T_{RR}}\overrightarrow{\tau}(t) dt\to 0$ and the OSI approximates its maximal value (OSI = 0.5).

## Results

### Phantom measurements

**Fig. 5:**
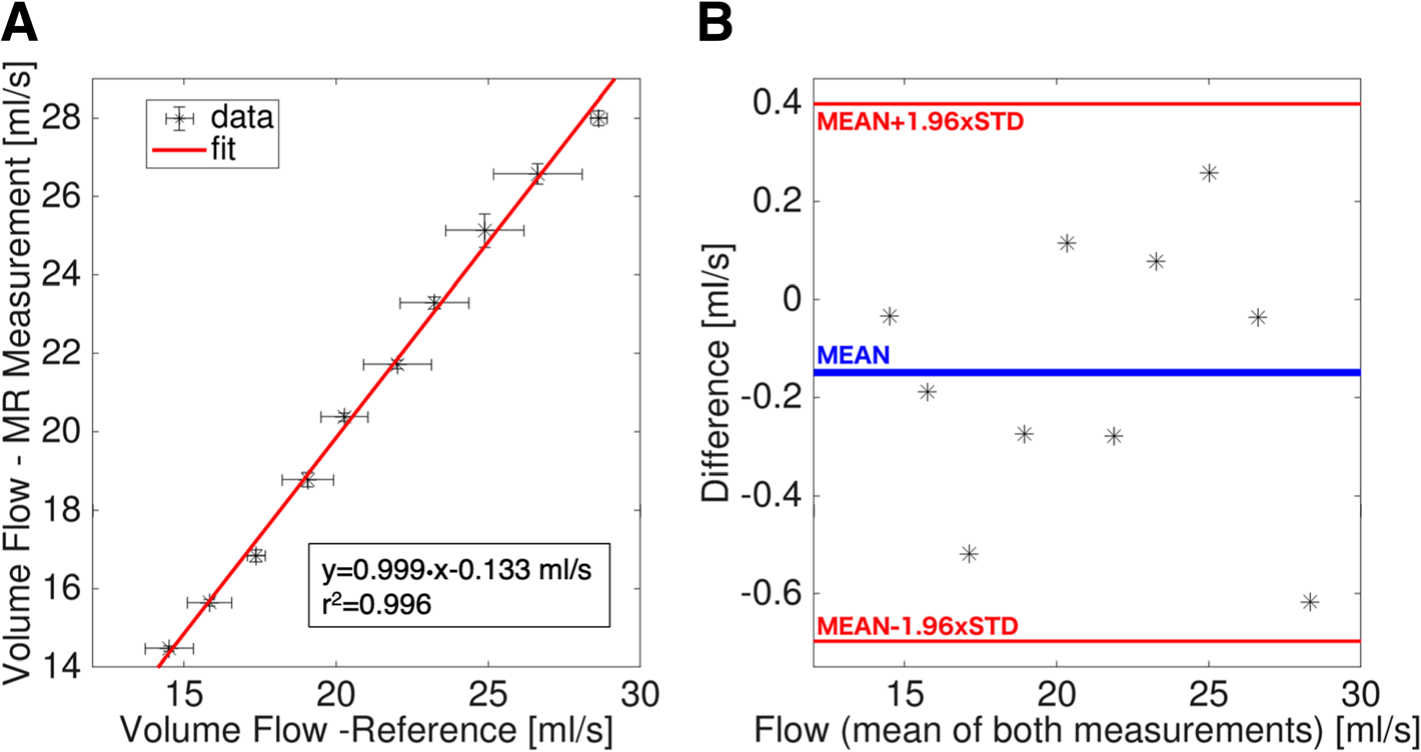
**A** Plot of the correlation between the flow values determined with the MR measurement and the volumetric measurement (both mean values and standard deviations). A high correlation coefficient (*r*^2^ = 0*.*996) could be derived. **B** Plot of the differences (between the MR measurement and the reference) against the mean values

Figure 5A shows a correlation plot between the CMR flow measurement and the reference values of the flow phantom measurements, indicating a high correlation (*r*^2^ = 0.996) between both methods. The Bland-Altman Plot in Fig. 5B reveals only a small bias of the CMR measurement relative to the reference (*−* 0*.*15 ml/s).

### Stability of self-navigation

**Fig. 6:**
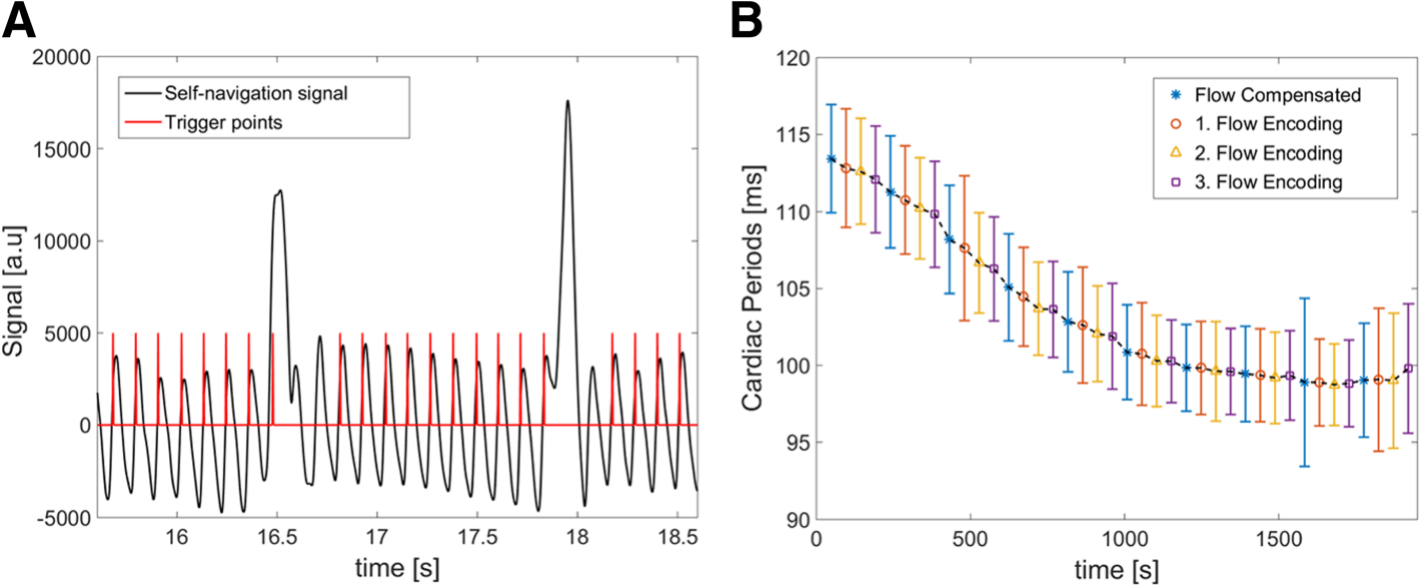
**A** Exemplary section of the self-navigation signal with the computed trigger points, measured in a representative wild-type mouse (1. flow-encoding measurement). The signal modulations due to cardiac and respiratory motions are clearly recognizable. **B** Mean values and variations of the cardiac periods for the 40 individual flow-encoding measurements (total measurement time: 32 min), obtained in the same mouse. Each data point represents an interval of 48 s. The mean variation of the cardiac periods found in this measurement was $\overline{\sigma}$ = *±*3.4 ms

Figure 6A shows an exemplary section of the self-navigation signal, measured in a representative mouse. The strong signal modulations due to cardiac and respiratory motion were used to calculate trigger points and breath-gating windows. To quantify the variance of the extracted trigger signals, mean values and variations of the cardiac periods were calculated for all individual measurements (Fig. 6B). No significant variations in quality between the 4 flow-encoding measurements were observed. The results of all 7 WT mice are displayed in Table 1. All animals showed a slight drift towards shorter cardiac periods during the measurement similar to Fig. 6B, which may be attributed to the adaption of the animal to the ambient temperature within the scanner and the anesthesia. However, the mean variation of the cardiac periods ($\overline{\sigma}$ ≤ 4.3 ms) indicates a high stability of the self-navigation signal during the whole measurement in all mice. Depending on the heart and respiratory rate, 15–20% of the data was discarded from the measurement due to respiration. Approximately 13,000–15,000 heart beats were detected in each measurement, providing about 6000–7500 spokes for each cine frame in the retrospective cine reconstruction.

**Table 1:** Range of cardiac periods (rr_min_-rr_max_) detected by the algorithm, mean variance and number of detected heart beats N for all 7 wild-type mice

Animal	rr_min_-rr_max_ [ms]	$\overline{\sigma}$ [ms]	N
*Mouse 1*	99–147	3*.*3	15,120
*Mouse 2*	93–129	3*.*4	15,680
*Mouse 3*	84–123	4*.*3	15,261
*Mouse 4*	96–141	2*.*7	13,328
*Mouse 5*	87–123	3*.*4	13,896
*Mouse 6*	99–135	4*.*2	13,057
*Mouse 7*	99–138	4*.*3	12,851

### Off-resonance correction

**Fig. 7:**
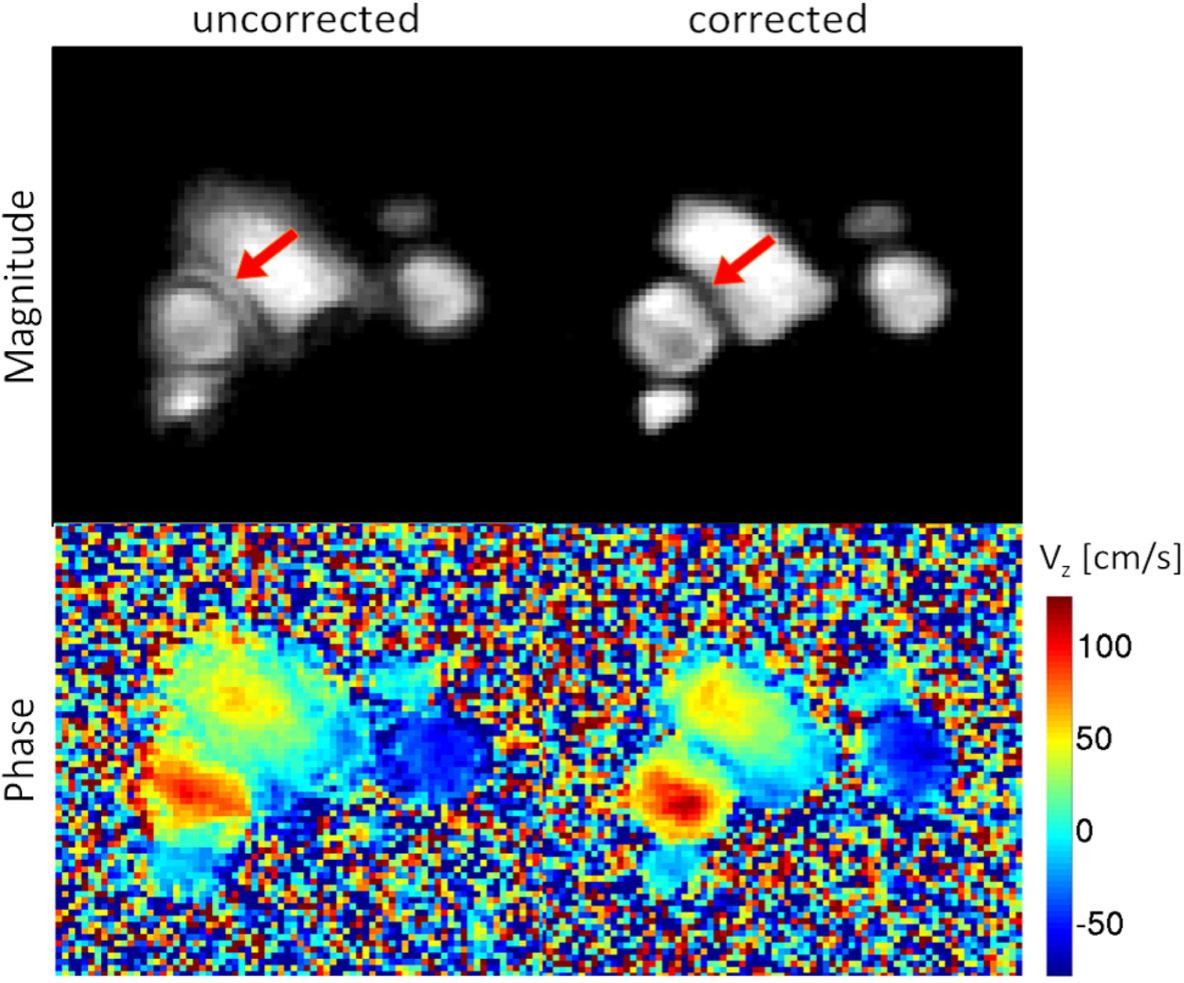
Magnitude images and maps of the *z*-velocity component (exemplary slice corresponding to a 3D dataset). Left: Without *B*_0_ correction. Right: After application of the *B*_0_ correction described above. The correction removes blurring and distortion artifacts

To demonstrate the effect of the off-resonance correction described above, magnitude images and velocity maps corresponding to the systolic cardiac phase are presented in Fig. 7. Uncorrected datasets result in severe blurring artifacts and distortions of the velocity data are recognizable (left), impeding further analysis of this data set. Using off-resonance correction, these artifacts can be removed (right).

### Flow and cross-sectional areas

**Fig. 8:**
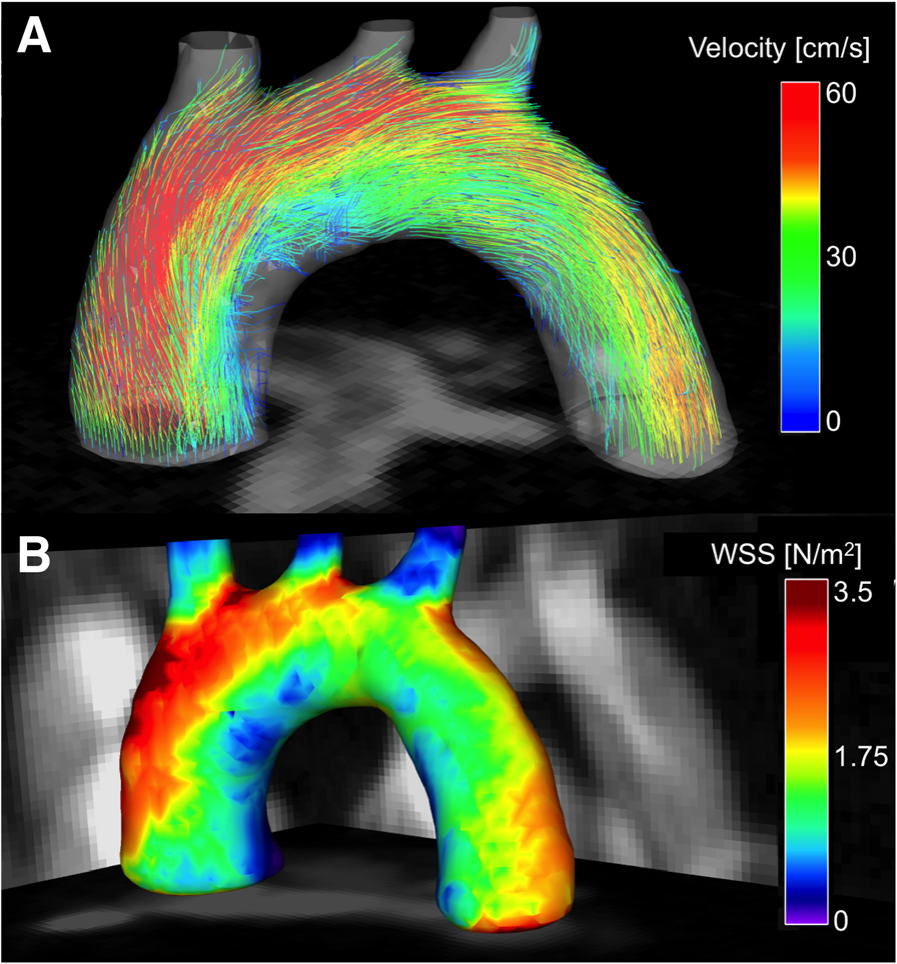
**A** Streamline visualization of the blood flow through the aortic arch during the systolic cardiac phase. The coloring of the streamlines indicates a strong asymmetric distribution of velocity values with the highest values near the arch’s outer radius. **B** Map of the time averaged WSS magnitude values, obtained from one exemplary mouse. In the background maximum intensity projections of the magnitude image are shown. The asymmetric distribution of velocity values leads into a large gradient of WSS values with the largest values near the outer radius of the aortic arch

To visualize the measured flow through the aortic arch, a streamline representation of velocities corresponding to the systolic cardiac phase is shown for an exemplary mouse in Fig. 8A. Due to the geometry of the arch, a strong asymmetric distribution of velocities is recognizable with the highest values near the outer radius. For further analysis, the maximum cross-sectional area (CSA) of the lumen, the peak volume flow and the net flow were assessed at 14 analysis planes along the aorta (Table 2). The results indicate a narrowing of the lumen relative to the ascending aorta in the bifurcation region and the descending aorta. CSA values of more than 2*.*1 mm^2^ were found in the ascending aorta. This corresponds to approximately 210 voxels and, given a circular geometry, to 16 voxels across the diameter. In the descending aorta, the area reduces to 1*.*2 mm^2^, which corresponds to 12 voxels across the diameter. Regarding the volume flow, a decline with increasing distance to the aortic root was observed, likely due to the partial outflow into the major branches.

**Table 2:** Peak cross-sectional areas (CSA), differences between maximum and minimum CSA, peak volume flow and netflow for 14 analysis planes (see Fig. 4B). All data are presented as average values over all 7 wild-type mice

Region	peak CSA [mm^2^]	CSA_max_-CSA_min_ [mm^2^]	peak flow [ml/s]	net flow [ml/s]
**Ascending aorta**				
1	1*.*96 *±* 0*.*30	0*.*49 *±* 0*.*08	0*.*88 *±* 0*.*11	4*.*09 *±* 0*.*77
2	2*.*00 *±* 0*.*24	0*.*51 *±* 0*.*07	0*.*90 *±* 0*.*11	4*.*22 *±* 0*.*66
3	2*.*06 *±* 0*.*20	0*.*56 *±* 0*.*06	0*.*90 *±* 0*.*10	4*.*20 *±* 0*.*58
4	2*.*08 *±* 0*.*14	0*.*55 *±* 0*.*04	0*.*87 *±* 0*.*10	4*.*06 *±* 0*.*45
5	2*.*10 *±* 0*.*11	0*.*53 *±* 0*.*05	0*.*83 *±* 0*.*09	4*.*01 *±* 0*.*36
6	2*.*14 *±* 0*.*10	0*.*52 *±* 0*.*09	0*.*80 *±* 0*.*09	3*.*92 *±* 0*.*41
**Bifurcation area**				
7	1*.*60 *±* 0*.*19	0*.*32 *±* 0*.*05	0*.*58 *±* 0*.*06	2*.*83 *±* 0*.*35
8	1*.*23 *±* 0*.*10	0*.*33 *±* 0*.*05	0*.*54 *±* 0*.*03	2*.*37 *±* 0*.*22
**Descending aorta**				
9	1*.*16 *±* 0*.*07	0*.*30 *±* 0*.*05	0*.*49 *±* 0*.*02	1*.*91 *±* 0*.*16
10	1*.*18 *±* 0*.*07	0*.*32 *±* 0*.*07	0*.*47 *±* 0*.*03	1*.*80 *±* 0*.*18
11	1*.*23 *±* 0*.*06	0*.*37 *±* 0*.*09	0*.*48 *±* 0*.*02	1*.*78 *±* 0*.*16
12	1*.*28 *±* 0*.*11	0*.*36 *±* 0*.*14	0*.*47 *±* 0*.*01	1*.*75 *±* 0*.*13
13	1*.*28 *±* 0*.*10	0*.*34 *±* 0*.*10	0*.*48 *±* 0*.*03	1*.*76 *±* 0*.*12
14	1*.*30 *±* 0*.*16	0*.*31 *±* 0*.*08	0*.*50 *±* 0*.*07	1*.*84 *±* 0*.*18

### Temporally averaged WSS

**Fig. 9:**
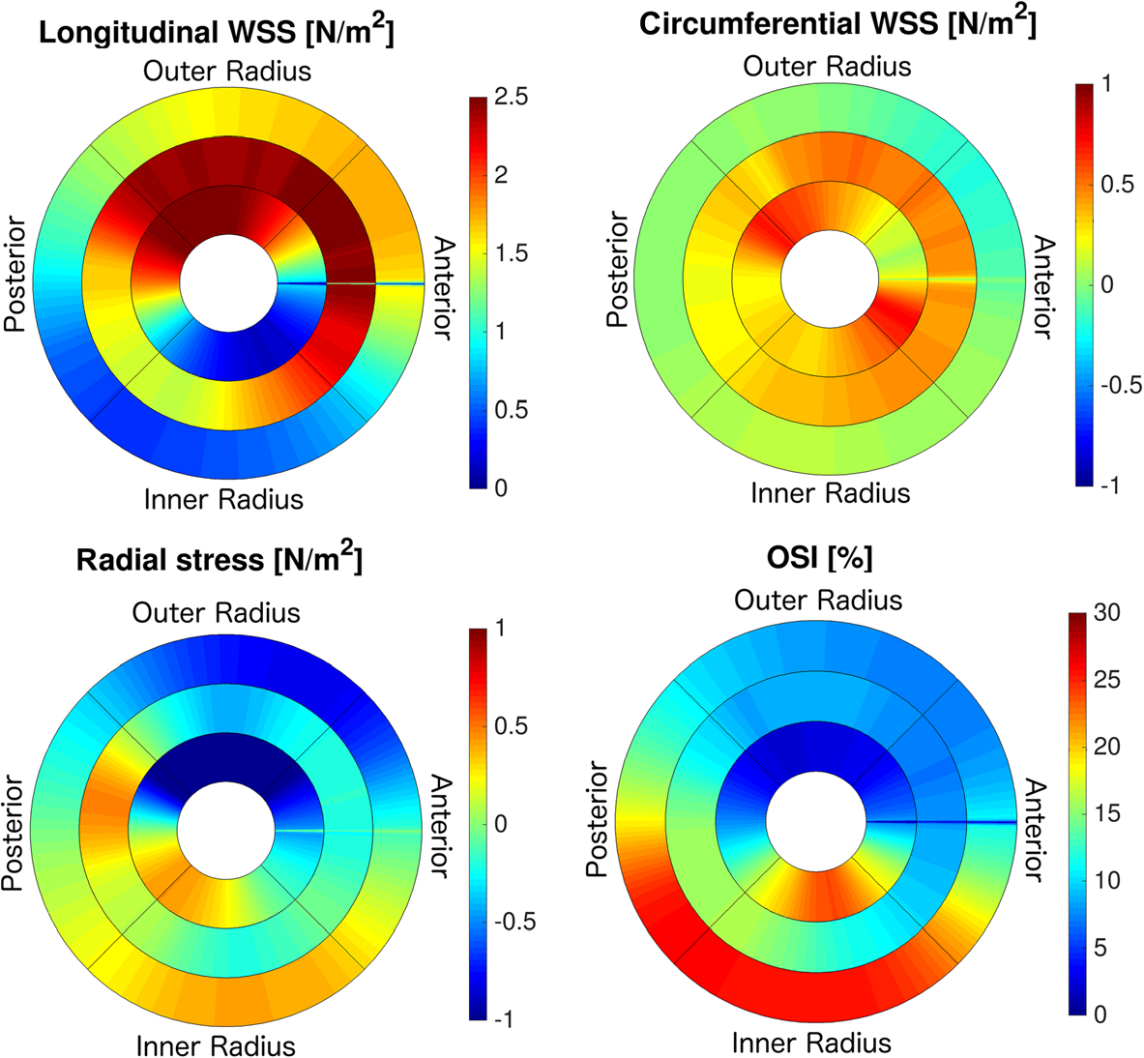
Bullseye-Plots for the distribution of the time-averaged longitudinal and circumferential WSS, radial stress and OSI (average over all 7 mice). The innermost circle represents the ascending aorta, the middle circle the bifurcation area and the outer circle the descending aorta

**Fig. 10:**
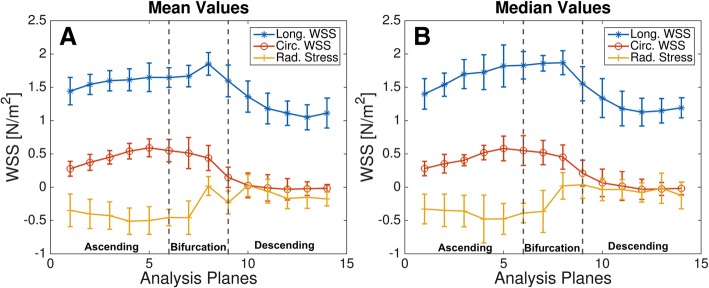
Distribution of the mean (**A**) and median (**B**) values of temporally averaged longitudinal, circumferential and radial stress components for 14 locations along the aortic arch. Analysis plane 1–6: Ascending aorta. 7–8: Bifurcation area. 9–14: Descending aorta. All data are presented as average values over all 7 wild-type mice

**Fig. 11:**
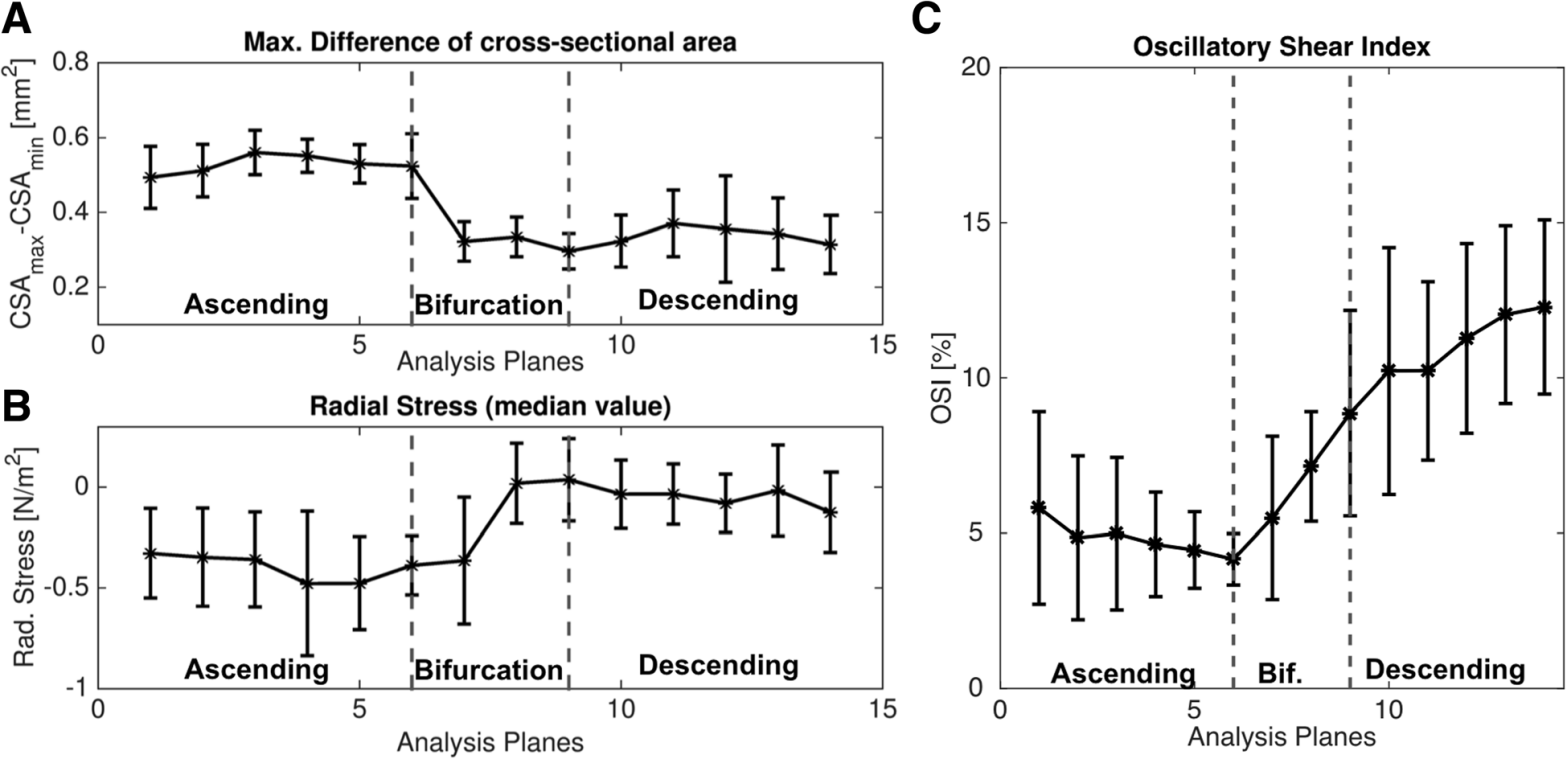
**A** Maximum differences between the cross sectional areas, CSA_max_-CSA_min_ and temporally averaged radial stress (**B**). An aligned behavior was observed between theses parameters. **C** Mean OSI values for all 14 analysis planes

Figure 8B shows a map of the temporally averaged WSS magnitude values in a representative mouse. In Fig. 9, Bullseye-plots are displayed to illustrate the distribution of the time-averaged longitudinal, circumferential and radial stress values over the vessel cross-section. As expected, the strong velocity gradient due to the aortic geometry leads to much larger longitudinal WSS values near the outer radius in comparison to the inner radius. Furthermore, radial stress has its maximum magnitude values near the outer radius of the ascending aorta. In Fig. 10 the profiles of mean and median values of the temporally averaged longitudinal, circumferential and radial stress components are shown for 14 locations along the aorta (see also Fig. 4B and Table 3). The mean longitudinal WSS increases with rising distance to the aortic root, reaching its maximum values at the top of the aorta (with bold emphasis in Table 3). This effect is even more pronounced for the median values (with bold emphasis in Table 3), indicating a strong asymmetric distribution of longitudinal WSS values in the ascending aorta. In the descending aorta, the longitudinal WSS declines, reaching its minimum value at plane 12–13. For the circumferential WSS, the maximum values were found at analysis plane 5 in the ascending aorta (with bold emphasis in Table 3). The circumferential WSS remains at high levels until analysis plane 8. In the descending aorta, a drop and even a sign change (planes 11–14) is observable. For the radial stress, the highest values were found in the ascending aorta near analysis plane 4. An aligned behavior was observed for the magnitude of radial stress values and the maximum changes of CSAs occurring during the cardiac cycle (see Table 2 and Fig. 11A).

**Table 3:** Mean and median values of the temporally averaged longitudinal, circumferential and radial stress for 14 locations along the aortic arch (see Fig. 4B). All data are presented as average values over all 7 wild-type mice

Region	long. WSS [N/m^2^]		circ. WSS [N/m^2^]		rad. Stress [N/m^2^]	
Ascending aorta	mean	median	mean	median	mean	median
1	1*.*44 *±* 0*.*21	1*.*40 *±* 0*.*23	0*.*28 *±* 0*.*11	0*.*28 *±* 0*.*11	*−* 0*.*35 *±* 0*.*25	*−* 0*.*33 *±* 0.22
2	1*.*54 *±* 0*.*15	1*.*54 *±* 0*.*17	0*.*37 *±* 0*.*11	0*.*35 *±* 0*.*14	*−* 0*.*40 *±* 0*.*22	*−* 0*.*35 *±* 0.24
3	1*.*60 *±* 0*.*15	1*.*70 *±* 0*.*22	0*.*45 *±* 0*.*12	0*.*40 *±* 0*.*08	*−* 0*.*43 *±* 0*.*21	*−* 0*.*36 *±* 0.24
4	1*.*61 *±* 0*.*17	1*.*73 *±* 0*.*26	0*.*54 *±* 0*.*10	0*.*52 *±* 0*.*11	**− 0.51 ± 0.20**	**− 0.48 ± 0.36**
5	1*.*65 *±* 0*.*21	1*.*82 *±* 0*.*32	**0.59 ± 0.13**	**0.58 ± 0.19**	*−* 0*.*50 *±* 0*.*21	*−* 0*.*48 *±* 0.23
6	1*.*65 *±* 0*.*15	1*.*83 *±* 0*.*21	0*.*55 *±* 0*.*17	0*.*55 *±* 0*.*22	*−* 0*.*46 *±* 0*.*12	*−* 0*.*39 *±* 0.15
Bifurcation. area	mean	median	mean	median	mean	median
7	1*.*67 *±* 0*.*16	1*.*86 *±* 0*.*12	0*.*51 *±* 0*.*23	0*.*52 *±* 0*.*18	*−* 0*.*46 *±* 0*.*25	*−* 0*.*36 *±* 0.31
8	**1.85 ± 0.17**	**1.87 ± 0.18**	0*.*44 *±* 0*.*19	0*.*45 *±* 0*.*18	0*.*02 *±* 0*.*14	0*.*02 *±* 0*.*20
Descending aorta	mean	median	mean	median	mean	median
9	1*.*60 *±* 0*.*24	1*.*55 *±* 0*.*26	**0.15 ± 0.15**	**0.21 ± 0.19**	−0.23 ± 0.17	*0.04 ± 0.20*
10	1*.*36 *±* 0*.*24	1*.*34 *±* 0*.*29	**0.03 ± 0.19**	**0.07 ± 0.21**	0.33 ± 0.16	−0.03 ± 0.7
11	1*.*18 *±* 0*.*23	1*.*18 *±* 0*.*26	**−0.01 ± 0.20**	**0.02 ± 0.22**	−0*.*06 *±* 0*.*18	*−*0*.*03 *±* 0.15
12	1*.*11 *±* 0*.*18	1*.*13 *±* 0*.*21	**−0.03 ± 0.16**	**−0.03 ± 0.15**	*−*0*.*17 *±* 0*.*15	*−*0*.*08 *±* 0.15
13	1*.*05 *±* 0*.*19	1*.*15 *±* 0*.*18	**−0.02 ± 0.10**	**−0.03 ± 0.10**	*−*0*.*15 *±* 0*.*16	*−*0*.*02 *±* 0.23
14	1*.*11 *±* 0*.*22	1*.*19 *±* 0*.*15	**−0.02 ± 0.06**	**−0.02 ± 0.10**	*−*0*.*18 *±* 0*.*10	*−*0*.*12 *±* 0.20

### Time resolved WSS and OSI

**Fig. 12:**
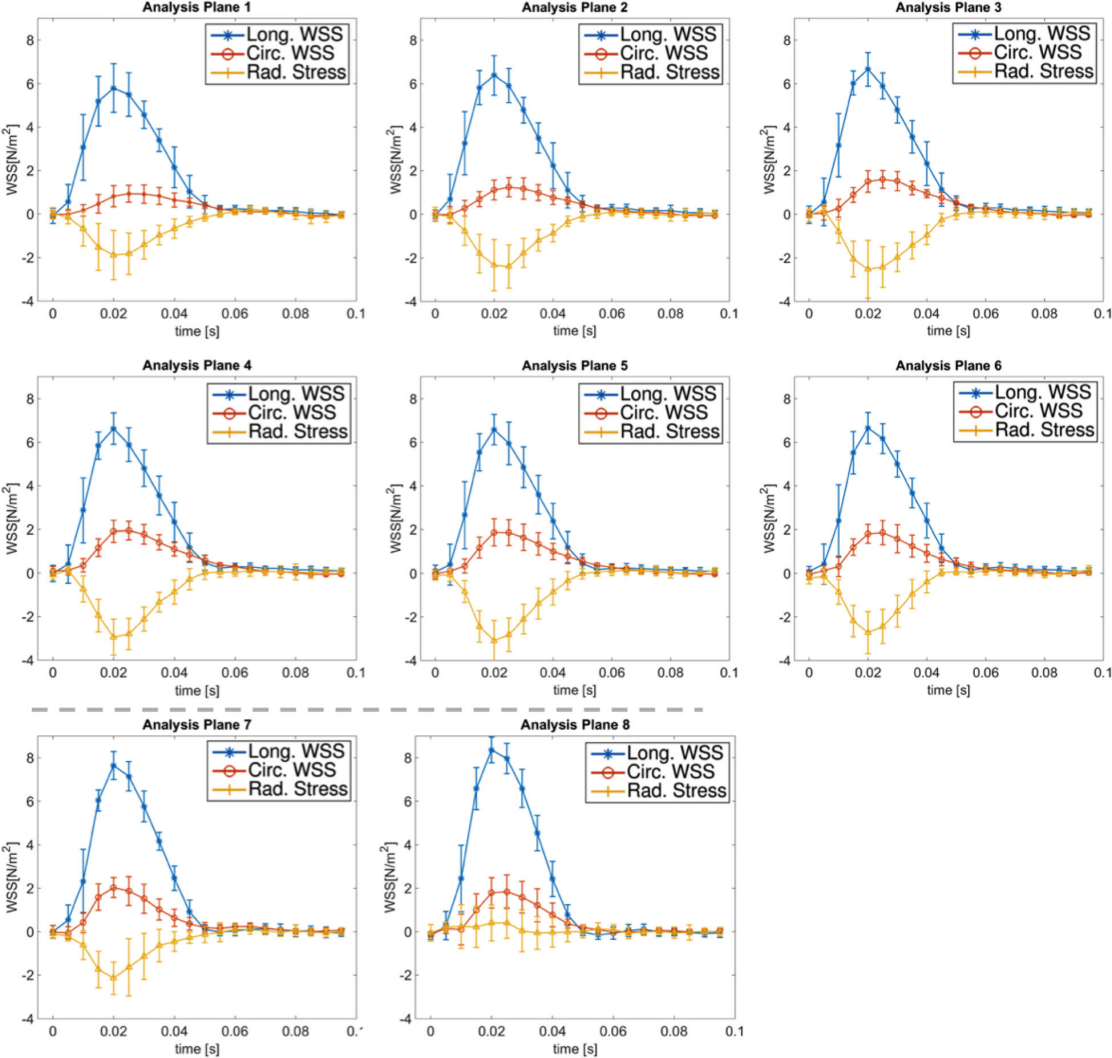
Time-resolved longitudinal, circumferential and radial stress values for the analysis planes 1–8 (ascending aorta and bifurcation area)

**Fig. 13:**
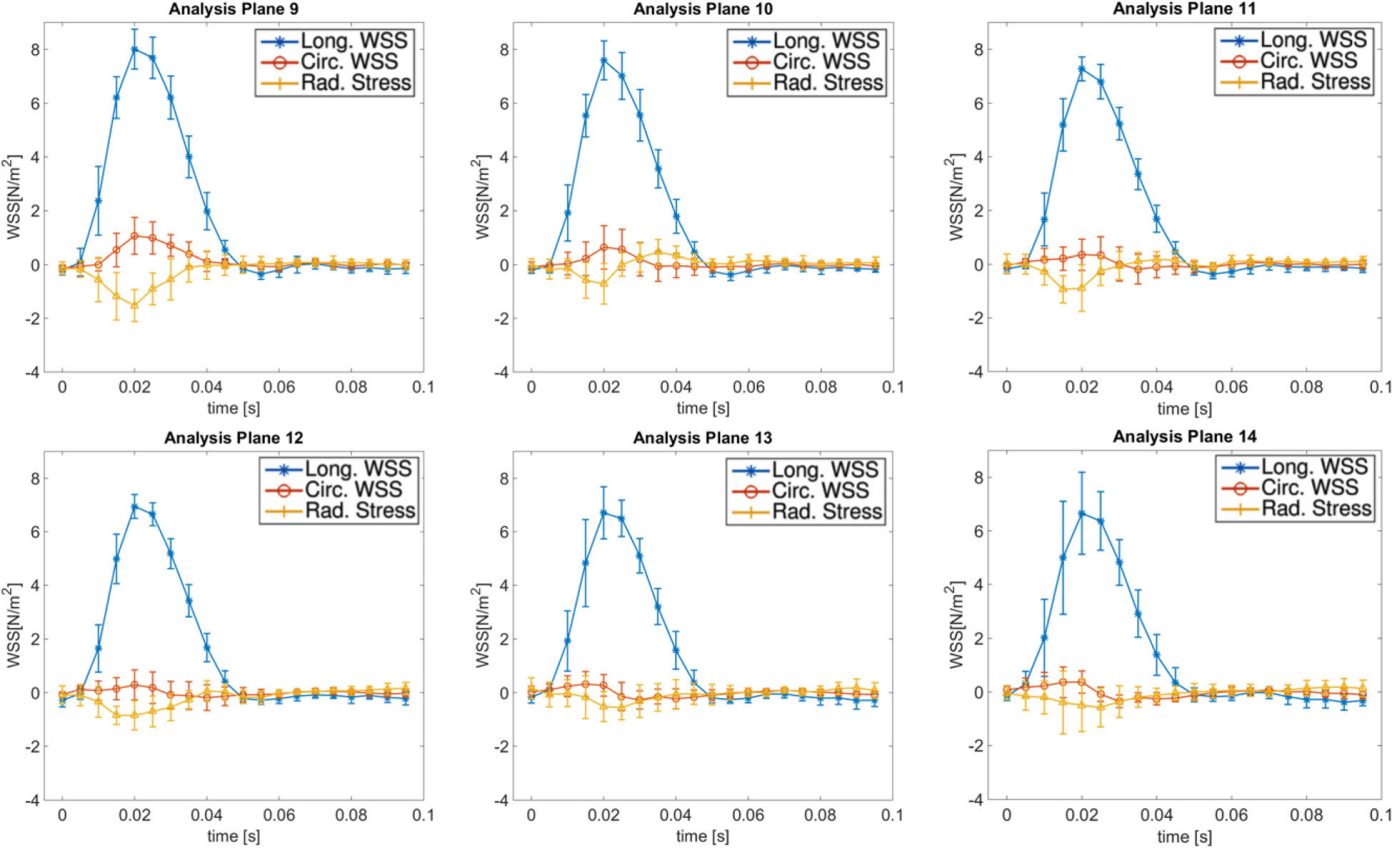
Time-resolved longitudinal, circumferential and radial stress values for the analysis planes 9–14 (descending aorta)

In Figs. 12 and 13 the time resolved mean longitudinal, circumferential and radial stress values are displayed for all 14 regions along the aorta. All three components show a strong pulsatile behavior during the systolic cardiac phase. In Table 4 the peak stress and OSI values are shown. The peak longitudinal WSS reaches its highest value at analysis plane 8 at the top region of the aorta, while the largest radial and circumferential WSS values can be found in plane 5 and 7, respectively (see with bold emphasis in Table 4). A drop of peak circumferential and radial stress values in the descending aorta is apparent. Regarding the OSI, the largest values were found near the inner radius of the ascending aorta where the longitudinal WSS has the lowest values (Fig. 9). Larger values were detected in the descending aorta (8.9–12.3%) compared to the ascending aorta (4.2–5.8%) (see Fig. 11B).

**Table 4:** Peak longitudinal, circumferential and radial stress and oscillatory shear index (OSI) for all 14 analysis planes (see Fig. 4B). All data are presented as average values over all 7 wild-type mice

Region	peak long. WSS [N/m^2^]	peak circ. WSS [N/m^2^]	peak rad. Stress [N/m^2^]	OSI [%]
Ascending aorta				
1	6*.*0 *±* 1*.*1	1*.*13 *±* 0*.*47	*−*2*.*2 *±* 1*.*1	5*.*8 *±* 3*.*1
2	6*.*59 *±* 0*.*93	1*.*39 *±* 0*.*49	*−*2*.*6 *±* 1*.*2	4*.*9 *±* 2*.*6
3	6*.*87 *±* 0*.*83	1*.*71 *±* 0*.*48	*−*2*.*8 *±* 1*.*2	5*.*0 *±* 2*.*5
4	6*.*90 *±* 0*.*66	2*.*08 *±* 0*.*47	*−* 3*.*20 *±* 0*.*92	4*.*6 *±* 1*.*7
5	6*.*82 *±* 0*.*68	2*.*01 *±* 0*.*60	**− 3.54 ± 0.96**	4*.*5 *±* 1*.*2
6	6*.*91 *±* 0*.*71	2*.*01 *±* 0*.*57	*−*3*.*3 *±* 1*.*0	4*.*2 *±* 0*.*83
Bifurcation area				
7	7*.*93 *±* 0*.*71	**2.38 ± 0.49**	*−*2*.*8 *±* 1*.*0	5*.*5 *±* 2*.*6
8	**8.62 ± 0.61**	2*.*10 *±* 0*.*76	*−*0*.*85 *±* 0*.*30	7*.*2 *±* 1*.*8
Descending aorta				
9	8*.*18 *±* 0*.*81	1*.*36 *±* 0*.*61	*−* 1*.*87 *±* 0*.*80	8*.*9 *±* 3*.*3
10	7*.*95 *±* 0*.*75	1*.*00 *±* 0*.*73	*−* 1*.*11 *±* 0*.*68	10*.*2 *±* 4*.*0
11	7*.*66 *±* 0*.*58	0*.*80 *±* 0*.*46	*−* 1*.*40 *±* 0*.*63	10*.*2 *±* 2*.*9
12	7*.*20 *±* 0*.*45	0*.*63 *±* 0*.*38	*−* 1*.*40 *±* 0*.*22	11*.*3 *±* 3*.*1
13	7*.*09 *±* 0*.*78	0*.*78 *±* 0*.*26	*−* 1*.*24 *±* 0*.*41	12*.*0 *±* 2*.*9
14	7*.*1 *±* 1*.*5	0*.*79 *±* 0*.*40	*−* 1*.*17 *±* 0*.*72	12*.*3 *±* 2*.*8

### Reproducibility

To test the reproducibility of the introduced method, 3 mice were measured twice within 7 days. Mean values and standard deviations (STD) of temporally averaged longitudinal and circumferential WSS as well as radial stress and OSI were calculated in 12 regions of interest (inner, outer, anterior and posterior radius in the ascending aorta, bifurcation area and descending aorta). Correlations between measurement 1 and 2, bias (mean of differences between measurement 1 and 2) and distribution (1*.*96 *×* STD of difference values) were determined with linear fitting and Bland-Altman plots (Table 5 and Additional file 1: Figures S1–S3). Reproducibility was found in particular for the longitudinal (*r*^2^ = 0*.*73–0*.*84) and circumferential (*r*^2^ = 0*.*63–0*.*74) WSS measurements.

**Table 5:** Reproducibility. Three mice were measured twice within 7 days. Longitudinal WSS, circumferential WSS and radial stress as well as OSI values were determined in 3 regions (ascending, bifurcation area, descending) and 4 sections, respectively. Correlation coefficient *r*^2^ (between measurement 1 and 2), bias (mean of difference between measurement 1 and 2) and scattering (1*.*96 SD of difference between measurement 1 and 2) were determined for all 4 measured variables. The corresponding plots can be found in Additional file 1: Figure S1–S3

Mouse	1	2	3
**Longitudinal WSS**			
Correlation *r*^2^	0*.*73	0*.*84	0*.*82
Bias [N/m^2^]	0*.*004	0*.*27	0*.*38
1*.*96*·* STD [N/m^2^]	0*.*807	0*.*65	0*.*53
**Circumferential WSS**			
Correlation *r*^2^	0*.*63	0*.*74	0*.*66
Bias [N/m^2^]	0*.*08	0*.*02	0*.*03
1*.*96*·* STD [N/m^2^]	0*.*43	0*.*65	0*.*50
**Radial Stress**			
Correlation *r*^2^	0.60	0*.*29	0*.*37
Bias [N/m^2^]	*−*0*.*11	0*.*21	*−*0*.*006
1*.*96*·* STD [N/m^2^]	0*.*82	0*.*65	1*.*38
**OSI**			
Correlation *r*^2^	0*.*72	0*.*22	0*.*15
Bias	*−*0*.*02	*−*0*.*04	*−*0*.*02
1*.*96*·* STD	0*.*07	0*.*15	0*.*10

### Subsampling

To investigate the influence of subsampling, one exemplary measurement (mouse 4 in Table 1) was reconstructed using 1, 2, 3 ... 10 subsets. Semiautomatic segmentation was performed for all 10 reconstructions. Mean values and standard deviations of temporally averaged longitudinal and circumferential WSS, radial stress and OSI were calculated for 12 regions of interest (see above) and all 10 reconstructions. The results are displayed in Additional file 1: Figures S4–S6. The strongest deviations relative to a full dataset are found when less than 6 subsets are used for reconstruction. To further assess the stability of the measurement and the measurement error, five 4D flow datasets were generated using different subsets (reconstruction 1: subsets 1–6, reconstruction 2: subsets 2–7, reconstruction 3: subsets 3–8, reconstruction 4: subsets 4–9, reconstruction 5: subsets 5–10). New lumen segmentations were generated by randomly combining the previous segmentations. Mean values and standard deviations were determined over 12 ROIs and the 5 datasets for all variables. The results are displayed in Table 6.

**Table 6:** Subsampling: Mean values and standard deviations, determined in 12 ROIs (ascending, top, descending with inner radius, outer radius, anterior radius, posterior radius, respectively) over 5 individual reconstructions in a representative mouse (mouse 4 in Table 1). Each 4D flow image was reconstructed from 6 subsets (reconstruction 1: subsets 1–6. reconstruction 2: subsets 2–7

Region	Longitudinal WSS [N/m^2^]	Circumferential WSS [N/m^2^]	Radial stress [N/m^2^]	OSI [%]
**Ascending aorta**				
inner radius	0*.*45 *±* 0*.*15	0*.*15 *±* 0*.*11	0*.*14 *±* 0*.*10	27*.*05 *±* 1*.*89
outer radius	2*.*48 *±* 0*.*09	0*.*51 *±* 0*.*03	*−*1*.*13 *±* 0*.*19	5*.*74 *±* 0*.*90
anterior radius	1*.*01 *±* 0*.*04	0*.*24 *±* 0*.*06	*−*0*.*63 *±* 0*.*14	5*.*22 *±* 4*.*09
posterior radius	1*.*97 *±* 0*.*08	0*.*19 *±* 0*.*09	0*.*12 *±* 0*.*09	7*.*92 *±* 4*.*39
**Bifurcation area**				
inner radius	1*.*26 *±* 0*.*07	0*.*05 *±* 0*.*04	*−*0*.*55 *±* 0*.*10	10*.*38 *±* 4*.*32
outer radius	2*.*31 *±* 0*.*14	0*.*94 *±* 0*.*15	*−*0*.*97 *±* 0*.*23	7*.*44 *±* 2*.*48
anterior radius	2*.*32 *±* 0*.*08	0*.*52 *±* 0*.*12	*−*0*.*73 *±* 0*.*10	3*.*41 *±* 2*,* 97
posterior radius	1*.*39 *±* 0*.*11	0*.*21 *±* 0*.*06	1*.*17 *±* 0*.*29	10*.*89 *±* 6*.*69
**Descending aorta**				
inner radius	0*.*47 *±* 0*.*06	*−*0*.*15 *±* 0*.*05	0*.*43 *±* 0*.*04	26*.*07 *±* 2*.*83
outer radius	1*.*50 *±* 0*.*07	*−*0*.*17 *±* 0*.*06	*−*0*.*93 *±* 0*.*09	8*.*35 *±* 1*.*62
anterior radius	1*.*50 *±* 0*.*07	*−*0*.*21 *±* 0*.*05	*−*0*.*30 *±* 0*.*05	16*.*05 *±* 2*.*00
posterior radius	1*.*11 *±* 0*.*02	0*.*01 *±* 0*.*02	0*.*05 *±* 0*.*05	13*.*35 *±* 4*.*97

## Discussion

### Measurement time

In this work we introduce a robust self-navigated technique for fast measurements of flow and wall shear stress in mice using radial PC-cine CMR. The acquisition time needed for a dataset with isotropic 100 μm resolution, including the additional *B*_0_ measurement, was only 35 min. This corresponds to an effective acceleration of almost 3 compared to a cartesian ECG-triggered 3D flow-encoding. Our investigations of the influence of subsampling indicate that an even further reduction of measurement time to approx. 20 min using only 6 subsets might be achievable. However, to guarantee the highest sampling density available for all measurements, reconstructions were always performed with 10 subsets in this study. The shorter scan time and the use of self-navigation instead of external trigger signals facilitates animal handling. The extra time gained due to the acceleration could be spent to expand the imaging protocol, e.g. for additional vessel wall morphology measurements. Further acceleration might be possible when combining the radial acquisition with *k − t* acceleration techniques [30]. In this study, images were reconstructed at a high spatial resolution (100 μm^3^) and a moderate temporal resolution (20 frames/cardiac cycle). The cine reconstruction framework can easily be adapted to also investigate high dynamic flow variations, e.g. by sacrificing spatial resolution while increasing the frame rate.

A radial 4D flow-encoding technique based on UTE-sampling and self-navigation was previously proposed in [15]. The main difference between the UTE-based flow- encoding technique and our proposed method is the longer measurement time and the smaller spatial resolution. Coverage of the full murine heart at an isotropic spatial resolution of 160 μm required a measurement time of 1 h 58 min. The longer scan protocol was due to the fact that global excitation was used in order to guarantee short echo times. The technique presented in this work, in contrast, uses a slice-selective excitation pulse and benefits from the strong signal suppression of the static tissue. Less data acquisition is required, hence the measurement time can be significantly reduced.

### Limitations

The proposed method strongly benefits from the ultrahigh magnetic field (17.6 T) and the high gradient strength (1 T/m), which allow high SNR values and short repetition times, both leading to shorter scan times. However, one limitation of the radial trajectory is its vulnerability to off-resonance effects, which can lead to severe blurring and distortion artifacts in the reconstructed magnitude images and velocity maps at ultrahigh field strengths. The first order *B*_0_ correction method presented in this work is computationally fast, easy to implement, and yields a significant improvement of image quality. However, in the lower part of the thoracic aorta close to the lung, large local field gradients can occur, which can lead to signal cancellations that cannot be reversed with the current method [30]. These artifacts lead to segmentation and phase errors, which can result in an underestimation of WSS values. However, with improved shimming and more advanced reconstruction techniques [32] it is feasible to reduce these artifacts. One further limitation is the susceptibility to signal cancellations caused by accelerated flow, which are more prominent at ultra high field strengths due to the larger local field gradients and especially present in the aortic root during the systolic cardiac phase. These artifacts can lead to an underestimation of flow values and hence to a possible underestimation of WSS values in the proximal part of the ascending aorta. In the presented method, we reduced flow artifacts by choosing a short TE (1.1 ms). We believe that it should be possible to reduce the vulnerability to flow by using ramp sampling [15] and slice-selective pulses for excitation. Furthermore the use of lower magnetic field strengths (7 T) in combination with cryogenic surface coils [10] should lead to a reduction of off-resonance and flow artifacts while still maintaining high SNR values.

### Flow and WSS values

Measurements of volume flow were conducted in a flow phantom and a group of 7 healthy WT mice. Both in vitro and in vivo measurements are in very good accordance with reference measurements and the literature [33–35].

WSS was directly derived by calculating the gradients of the measured 3D velocity field at the segmented lumen surface. As shown previously with a 3D spiral sequence in mice [7], a strong asymmetric distribution, yielding the highest values near the outer radius and the lowest values near the inner radius of the aortic arch, could be observed. Longitudinal and circumferential components of the temporally averaged WSS as well as radial stress were calculated at 14 sites along the aorta. The highest longitudinal WSS values were found in the top region of the arch, which is in accordance with the results reported previously [7]. In the descending aorta, the temporal averaged WSS values are in range with the values reported previously for 9-month-old WT mice (0.8–2.1 N/m^2^, see [9]). The lowest or even negative longitudinal WSS were found near the inner radius of the ascending aorta and the bifurcation area near the aortic branches, indicating low or even recirculative flow near these regions. For the distribution of mean circumferential WSS, a similar behavior was observed as reported for studies in humans [5, 8] and mice [7], yielding the highest values in the upper ascending aorta and the top region of the arch.

Investigations of repeatability showed that the measurement of both WSS components can be well reproduced. Possible causes of deviations might be differences in slice positioning, uncertainties in calculating an accurate centerline and *B*_0_ inhomogeneities. Overall, WSS values were larger than reported previously for 6-months old ApoE^*−/−*^ mice [7] but still lower than reported for measurements using computational fluid dynamics for WSS calculation, most likely due to the still low spatial resolution [36]. Further improvements of the described technique should include the use of interpolation and CFD in order to reduce this error.

In 2017, Braig et al. presented an ECG-triggered cartesian sequence for preclinical measurements of the WSS in the murine aortic arch of 4-weeks-old WT mice [10]. The sequence enables 4D flow measurements within 40 min, however, the native spatial resolution was much lower (300 μm) and the evaluation workflow only considered the longitudinal WSS without taking into account circumferential WSS. Furthermore the method presented in this paper still required an ECG signal for navigation while our method enables completely wireless WSS measurements. Peak flow values were similar to those observed in our study (see Table 2). Regarding the peak longitudinal WSS, we measured higher values (6–8 N/m^2^) relative to the results presented by Braig et al. (4–6 N/m^2^), which might be due to the higher spatial resolution and the difference in age. Furthermore, we also observed an increase of the peak longitudinal WSS with rising distance from the aortic root. In accordance with the previous study, the highest peak longitudinal values were measured in the top region between the 2nd and 3rd bifurcation (see Figs. 12 and 13).

As byproduct of the calculation of the stress tensor, radial components pointing towards the vessel wall could be derived. An aligned relationship between the distribution of radial stress and the dilatation of aorta occurring during the cardiac cycle was observed. Since aortic stiffness has a large influence on the degree of aortic dilatation, the investigation of possible correlations between the magnitude of radial stress and the elasticity could be interesting. To our knowledge this is the first time that results for the radial component are reported for mice. However, since the radial stress values could not be as well reproduced, the true benefit of this parameter still needs to be investigated.

### OSI

Regarding the OSI, the largest values were measured in the upper part of the descending aorta, indicating a larger amount of oscillatory and recirculative flow in these areas. The OSI has its highest values near the inner radius of the aortic arch, which is in agreement with the results reported for ApoE^*−/−*^ mice [7]. OSI values were between 4*.*6% (ascending aorta) and 12*.*3% (descending aorta). Since the OSI usually has its highest values in regions where the WSS magnitude is low, the measurement of this parameter is more susceptible to SNR and could not be as well reproduced as the longitudinal and circumferential WSS measurements. The results, however, closely correspond with the values found in literature [7].

## Conclusion

In summary, a robust accelerated measurement of flow and wall shear stress in the murine aortic arch was presented. The new method does not require ECG triggering and enables easier animal handling. The longitudinal, circumferential and radial component of the WSS and OSI values could be assessed. Future studies should focus on WSS measurements in atherosclerotic mouse models and possible correlations with pulse wave velocities and vessel wall morphology.

## Additional file

Additional file 1: **Figure S1–S3.** Reproducibility of the measurement method, tested in three different mice at two measurement days (time between two measurement days: 7 days). Correlation and Bland-Altman Plots were generated for all relevant parameters. In particular for the longitudinal and circumferential WSS values, a good reproducibility can be observed. Deviations between both measurements are mainly caused by differences in slice positioning, uncertainties in calculating the centerline and segmentation as well as off-resonance effects. **Figure S4–S6.** Stability of the measurement method, assessed by reconstructing one dataset using varying numbers of subsets (*n* = 1, 2, . . ., 10) for reconstruction. The largest deviation (relative to the 10-subsets-reconstruction) was found, when less than 6 subsets were used for reconstruction. To assess the error within a measurement, 5 reconstructions were generated using different ensembles of subsets (reconstruction 1: subsets 1–6. reconstruction 2: subsets 2–7. reconstruction 3: subsets 3–8. reconstruction 4: subsets 4–9. reconstruction 5: subsets 5–10). Mean values and standard deviations were determined over all 5 datasets for 12 regions of interests. For the longitudinal WSS, a mean standard deviation of approx. 7.5% was estimated. (PDF 16066 kb)

## Data Availability

Please contact author for data requests.

## Abbreviations

ApoE: Apolipoprotein E
CMR: Cardiovascular magnetic resonance
CSA: Cross-sectional area
ECG: Electrocardiogram
FLASH: Fast Low Angle Shot
MRI: Magnetic Resonance Imaging
NUFFT: Nonuniform Fast Fourier Transform
OSI: Oscillatory Shear Index
PC: Phase contrast
ROI: Region of interest
SNR: Signal to noise ratio
TEM: Transmit-receive electromagnetic
TOF: Time-of-flight
UTE: Ultrashort echo time
WSS: Wall Shear Stress
WT: Wild-type
