# Supplementary figures and images for: Fast self-navigated wall shear stress measurements in the murine aortic arch using radial 4D-phase contrast cardiovascular magnetic resonance at 17.6 T
Source: J Cardiovasc Magn Reson. 2019 Oct 14;21:64. doi: 10.1186/s12968-019-0566-z (PMC6792269; doi:10.1186/s12968-019-0566-z)

# Figure S1

**A**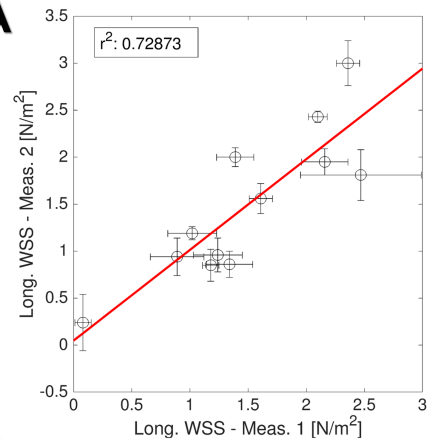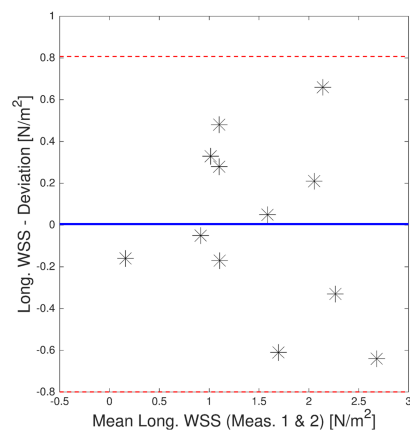**B**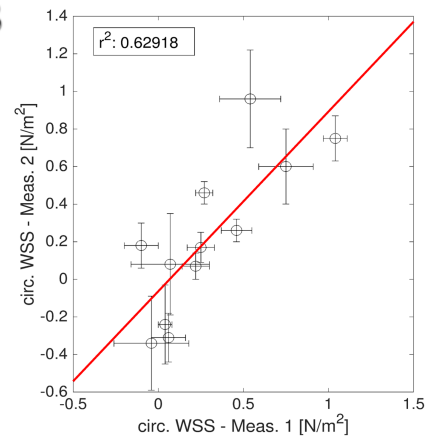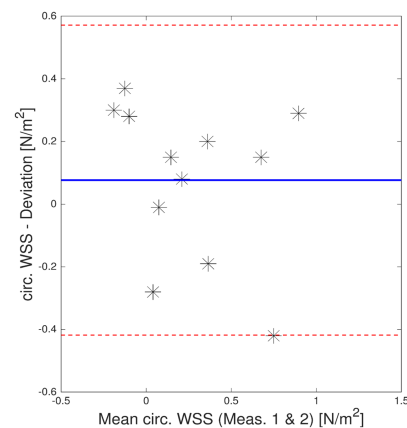**C**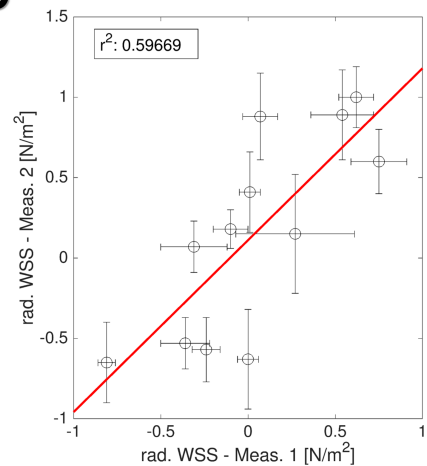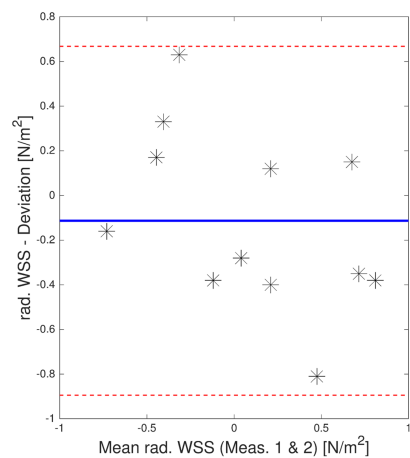**D**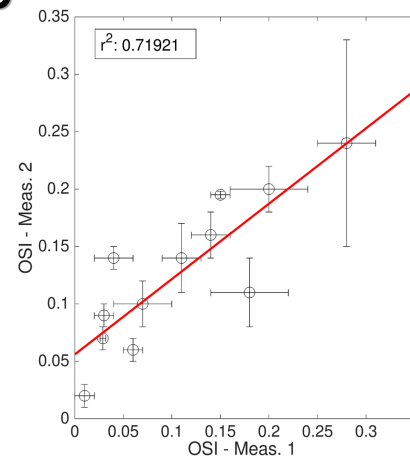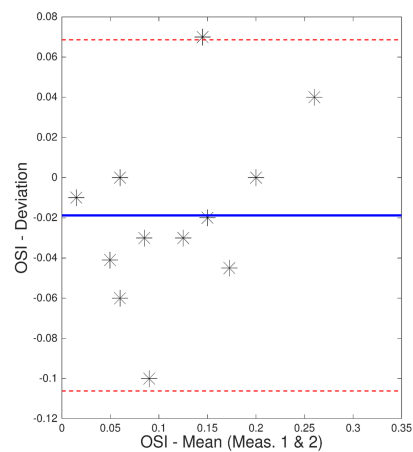

# Figure S2

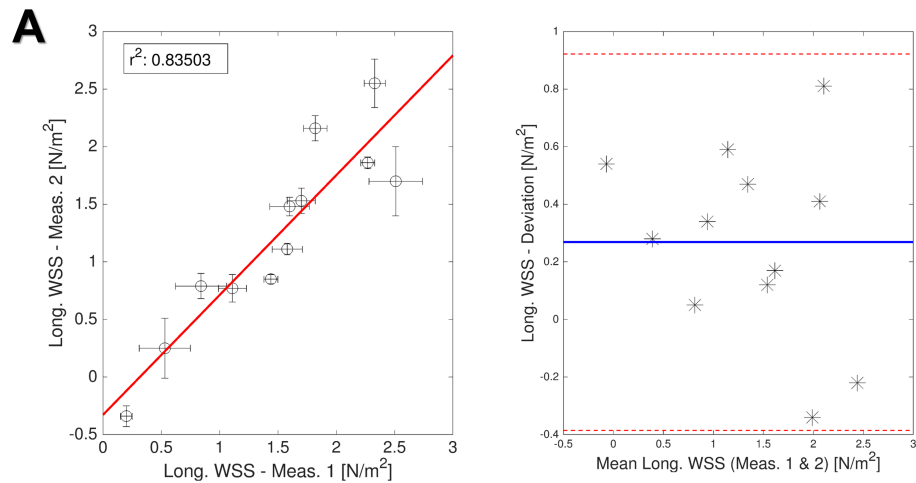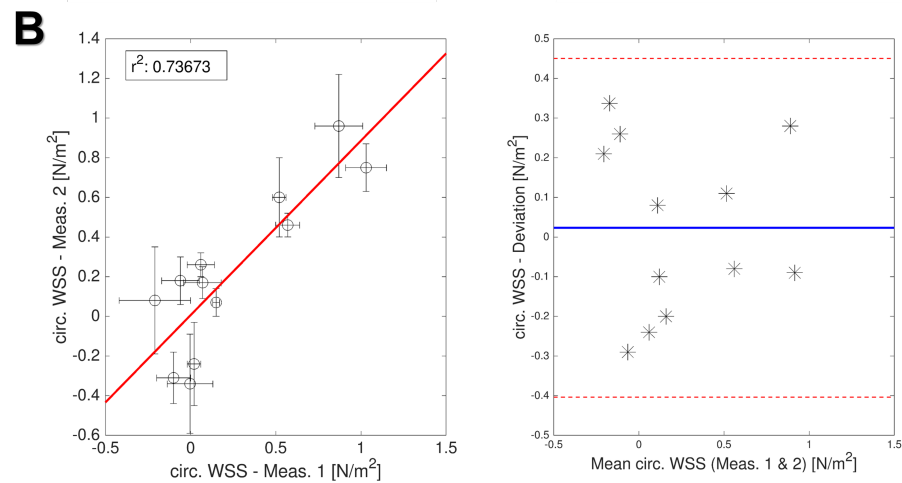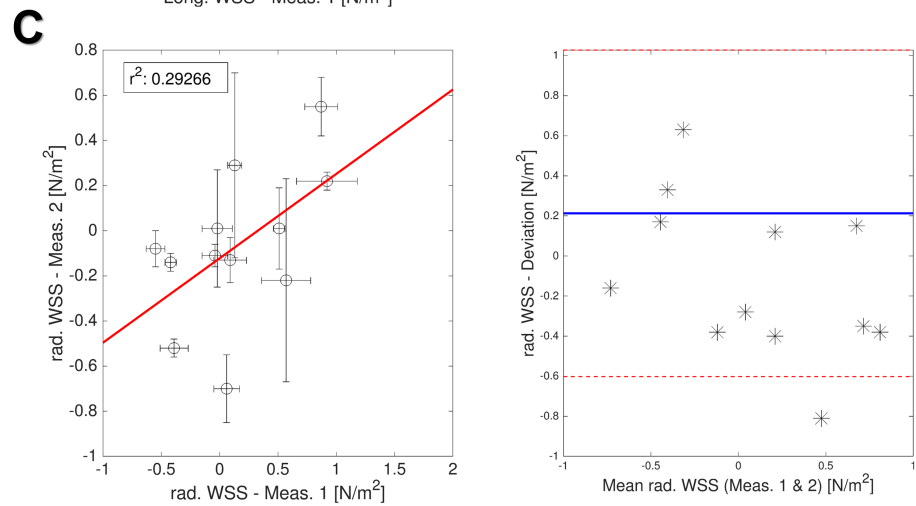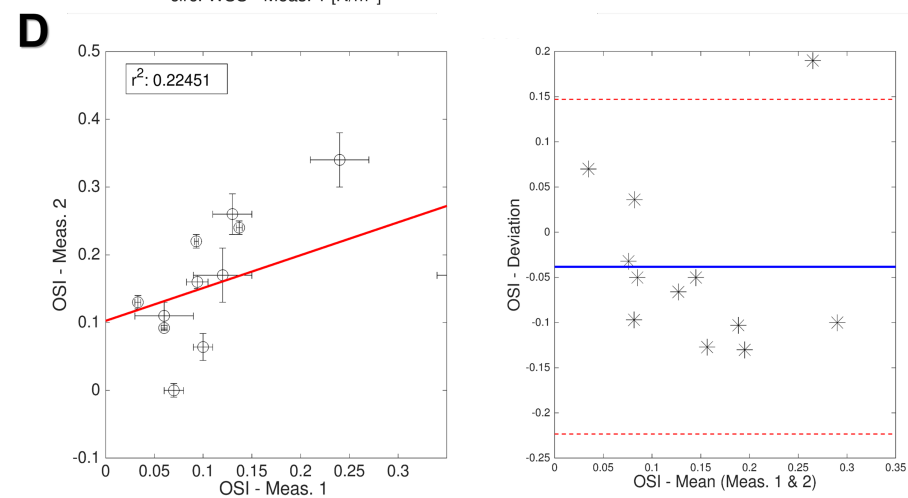

# Figure S3

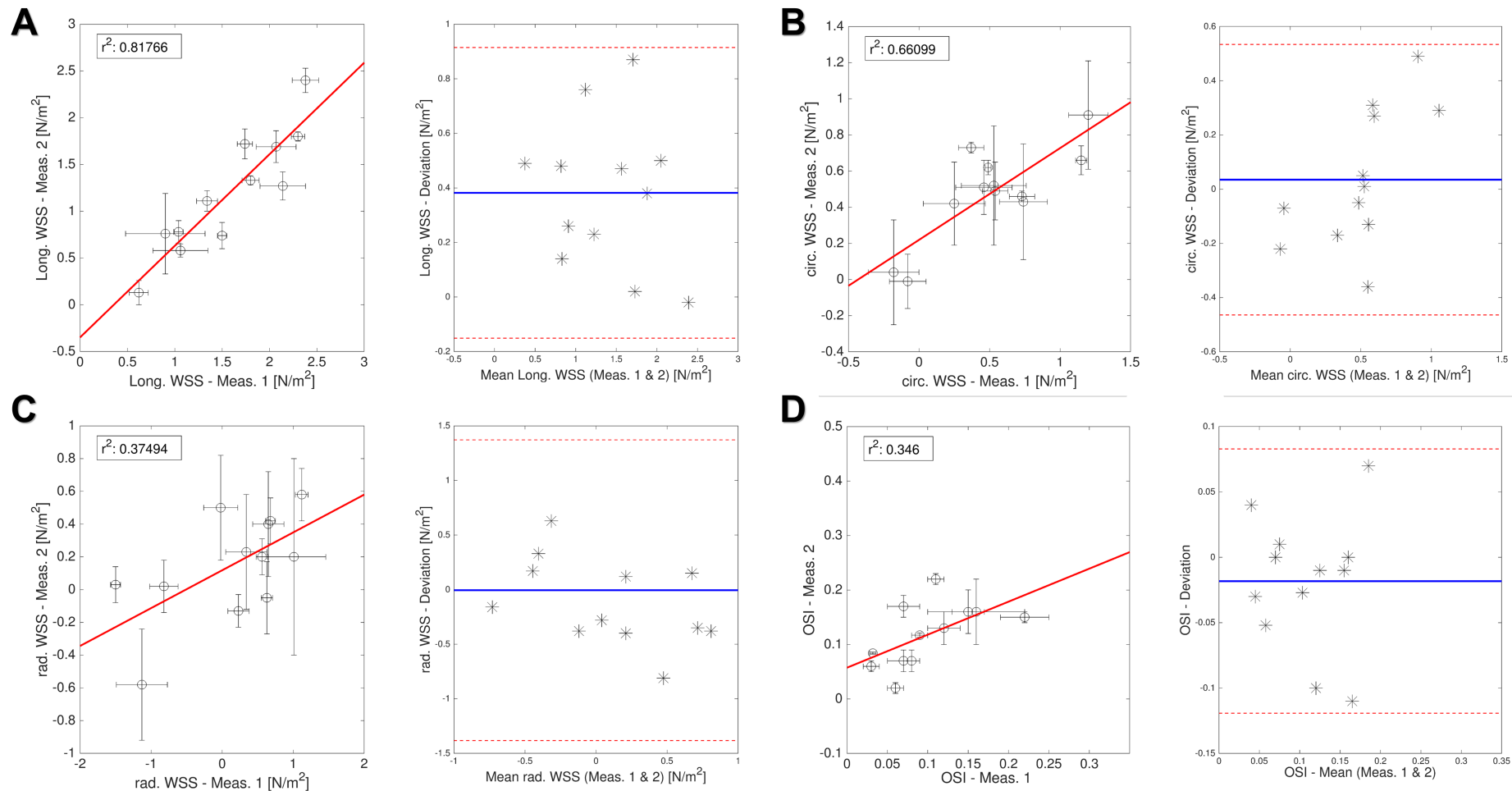

# Figure S4

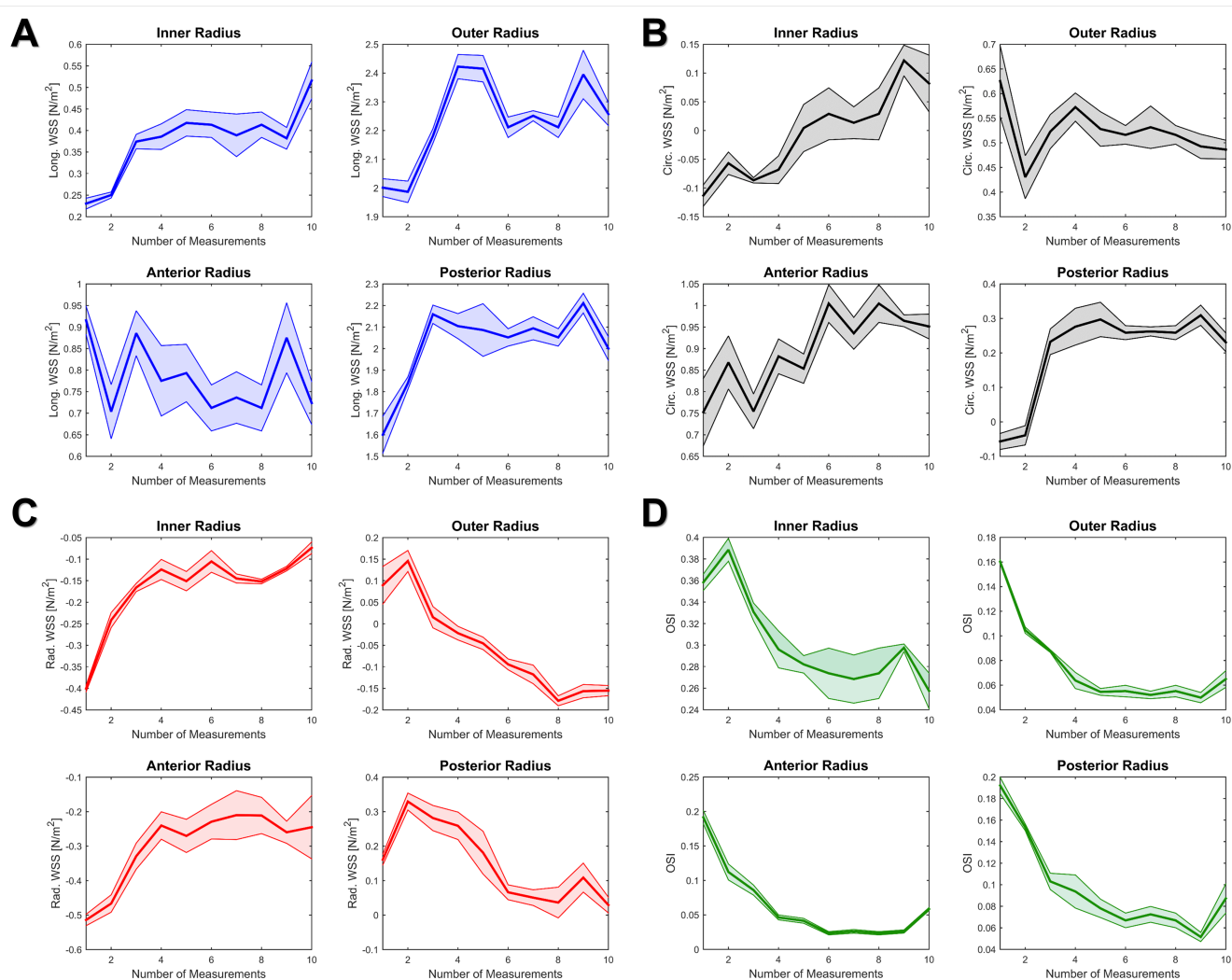

# Figure S5

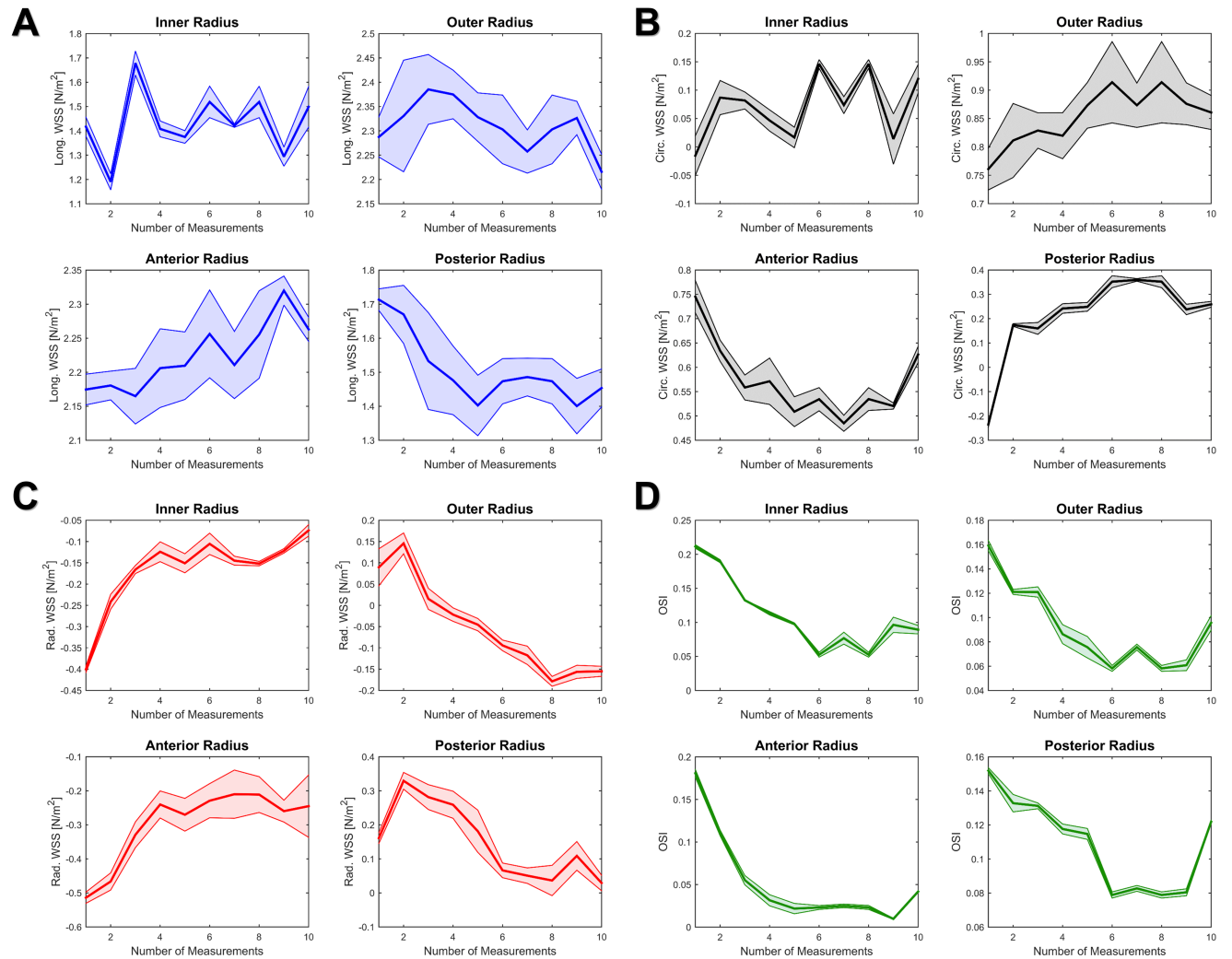

# Figure S6

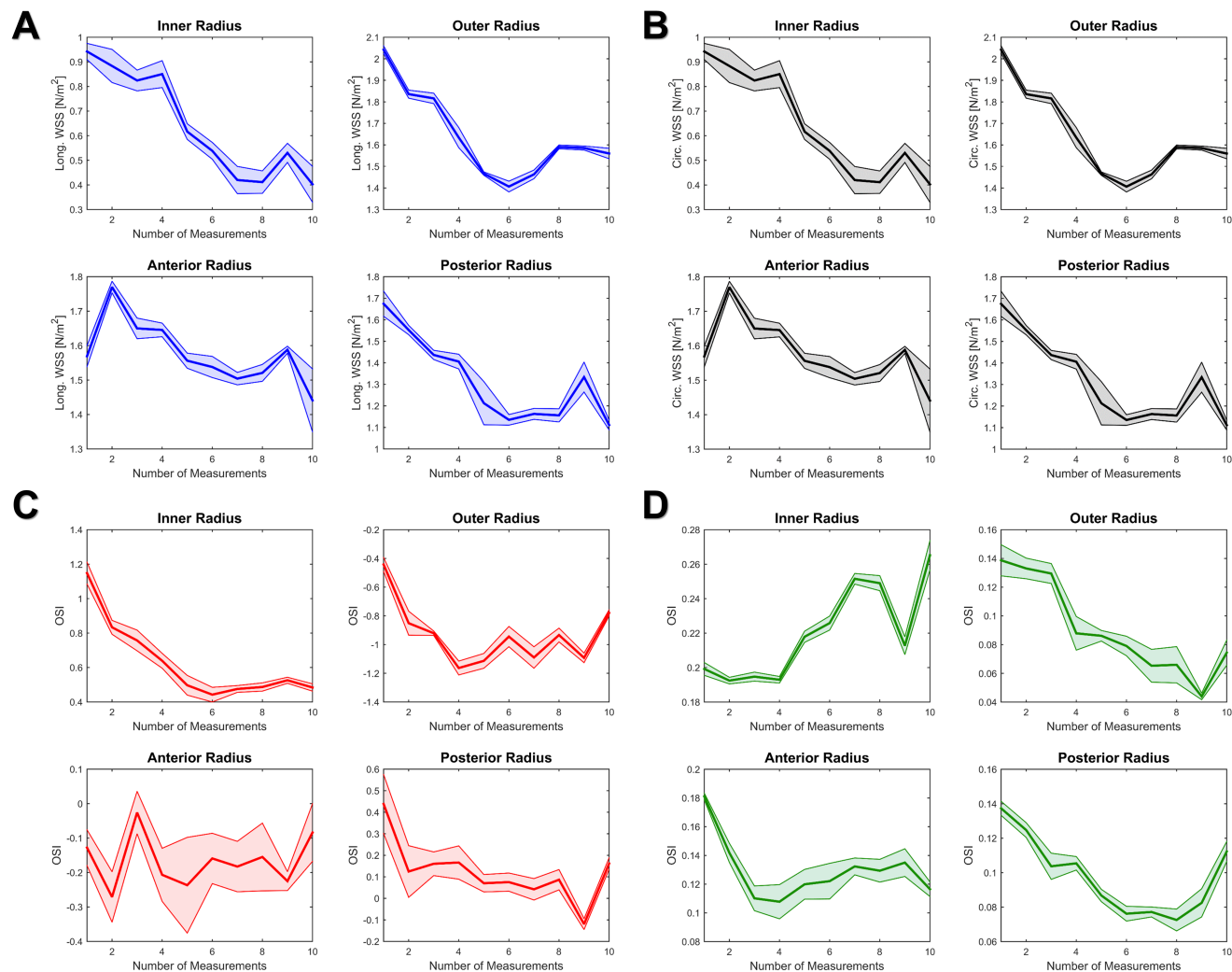

Supplement: Supplementary file 1 — Figure S1–S3. Reproducibility of the measurement method, tested in three different mice at two measurement days (time between two measurement days: 7 days). Correlation and Bland-Altman Plots were generated for all relevant parameters. In particular for the longitudinal and circumferential WSS values, a good reproducibility can be observed. Deviations between both measurements are mainly caused by differences in slice positioning, uncertainties in calculating the centerline and segmentation as well as off-resonance effects. Figure S4–S6. Stability of the measurement method, assessed by reconstructing one dataset using varying numbers of subsets (n = 1, 2, . . ., 10) for reconstruction. The largest deviation (relative to the 10-subsets-reconstruction) was found, when less than 6 subsets were used for reconstruction. To assess the error within a measurement, 5 reconstructions were generated using different ensembles of subsets (reconstruction 1: subsets 1–6. reconstruction 2: subsets 2–7. reconstruction 3: subsets 3–8. reconstruction 4: subsets 4–9. reconstruction 5: subsets 5–10). Mean values and standard deviations were determined over all 5 datasets for 12 regions of interests. For the longitudinal WSS, a mean standard deviation of approx. 7.5% was estimated. (PDF 16066 kb) [file 12968_2019_566_MOESM1_ESM.pdf]
